# Yeast Screens Identify the RNA Polymerase II CTD and SPT5 as Relevant Targets of BRCA1 Interaction

**DOI:** 10.1371/journal.pone.0001448

**Published:** 2008-01-16

**Authors:** Craig B. Bennett, Tammy J. Westmoreland, Carmel S. Verrier, Carrie A. B. Blanchette, Tiffany L. Sabin, Hemali P. Phatnani, Yuliya V. Mishina, Gudrun Huper, Alice L. Selim, Ernest R. Madison, Dominique D. Bailey, Adebola I. Falae, Alvaro Galli, John A. Olson, Arno L. Greenleaf, Jeffrey R. Marks

**Affiliations:** 1 Department of Surgery, Duke University Medical Center, Durham, North Carolina, United States of America; 2 Department of Medicine, Duke University Medical Center, Durham, North Carolina, United States of America; 3 Department of Biochemistry, Duke University Medical Center, Durham, North Carolina, United States of America; 4 Gene and Molecular Therapy Laboratory, Institute of Clinical Physiology, Consiglio Nazionale delle Ricerche (CNR), CNR Research Area Via Moruzzi, Pisa, Italy; Northwestern University, United States of America

## Abstract

BRCA1 has been implicated in numerous DNA repair pathways that maintain genome integrity, however the function responsible for its tumor suppressor activity in breast cancer remains obscure. To identify the most highly conserved of the many BRCA1 functions, we screened the evolutionarily distant eukaryote *Saccharomyces cerevisiae* for mutants that suppressed the G1 checkpoint arrest and lethality induced following heterologous BRCA1 expression. A genome-wide screen in the diploid deletion collection combined with a screen of ionizing radiation sensitive gene deletions identified mutants that permit growth in the presence of BRCA1. These genes delineate a metabolic mRNA pathway that temporally links transcription elongation (*SPT4, SPT5, CTK1, DEF1*) to nucleopore-mediated mRNA export (*ASM4, MLP1, MLP2, NUP2, NUP53, NUP120, NUP133, NUP170*, *NUP188, POM34*) and cytoplasmic mRNA decay at P-bodies (*CCR4, DHH1*). Strikingly, BRCA1 interacted with the *p*hosphorylated RNA polymerase II (RNAPII) *c*arboxy *t*erminal *d*omain (P-CTD), phosphorylated in the pattern specified by the CTDK-I kinase, to induce *DEF1*-dependent cleavage and accumulation of a RNAPII fragment containing the P-CTD. Significantly, breast cancer associated BRCT domain defects in BRCA1 that suppressed P-CTD cleavage and lethality in yeast also suppressed the physical interaction of BRCA1 with human SPT5 in breast epithelial cells, thus confirming SPT5 as a relevant target of BRCA1 interaction. Furthermore, enhanced P-CTD cleavage was observed in both yeast and human breast cells following UV-irradiation indicating a conserved eukaryotic damage response. Moreover, P-CTD cleavage in breast epithelial cells was BRCA1-dependent since damage-induced P-CTD cleavage was only observed in the mutant BRCA1 cell line HCC1937 following ectopic expression of wild type BRCA1. Finally, BRCA1, SPT5 and hyperphosphorylated RPB1 form a complex that was rapidly degraded following MMS treatment in wild type but not BRCA1 mutant breast cells. These results extend the mechanistic links between BRCA1 and transcriptional consequences in response to DNA damage and suggest an important role for RNAPII P-CTD cleavage in BRCA1-mediated cancer suppression.

## Introduction

Inherited heterozygous BRCA1 and BRCA2 defects have been associated with an enhanced risk of early onset breast cancer. These tumor suppressor genes normally function to maintain genome integrity by participating in the repair of DNA damage. Although the repair-related functions of BRCA1 have been extensively characterized (reviewed in [1]), identifying the underlying mechanism responsible for the onset of breast cancer has remained elusive. The BRCA1 protein functions in multiple pathways that maintain genomic integrity through its impact on the repair of a variety of genomic lesions including DNA double-strand breaks (DSBs). BRCA1 participates in recombinational repair of DSBs, non-homologous end-joining of DSBs, checkpoint arrest, transcriptional regulatory mechanisms and chromatin remodeling as well as centrosome duplication [1] [2]. Moreover, the BRCA1 protein has been found to physically interact with numerous other proteins in distinct, multi-component DNA repair complexes [3] [4] [5]. Although BRCA1 seems to be a central DNA repair component due to its many physical interactions in repair-related processes, the tumor suppressor activity is largely confined to breast and ovarian epithelia [1]. To resolve this enigma, BRCA1 has been suggested to interact with a subset of tissue specific, non-redundant cofactors in a single key pathway to mediate its tumor suppressive effects.

As an alternative approach for studying this important repair protein, we have utilized the yeast *Saccharomyces cerevisiae,* which has served as the fundamental model organism for the identification of the genetic controls associated with DNA repair and checkpoint functions that are conserved in most eukaryotes [6]. Yeast is an excellent model organism for elucidating BRCA1 function since heterologous expression of BRCA1 causes slow growth and lethality in both haploid [7] and diploid [8] wild type (WT) yeast. Mutations within the BRCT domain of BRCA1 that are associated with familial breast cancer, but not polymorphisms without disease association, suppress this slow growth phenotype [7].

In order to identify a key pathway that may underlie the tumorigenic potential of BRCA1 in breast cancer, we took advantage of the evolutionary distance between humans and yeast. In effect, we used yeast as an evolutionary “filter” to identify conserved proteins that, through severe functional constraints, have maintained the ability to interact with BRCA1 over a vast evolutionary distance. Other successful examples of this approach include the identification of relevant conserved genetic and biochemical networks that interact with mutant Huntington protein as well as alpha-synuclein, which has been implicated in the pathology of Parkinson's disease [9]; [10]. Since interaction of BRCA1 with such conserved proteins may account for lethality in WT yeast, we screened for nonessential gene deletions in the yeast diploid deletion collection that suppressed BRCA1-induced lethality. Previously, we described that deletions of the G1 checkpoint adaptation genes *CCR4* or *DHH1* that also mediate radiation resistance, suppressed BRCA1-induced lethality [8].

In this report we screened both ionizing radiation (IR) sensitive mutants and an unselected pool of yeast deletion mutants to identify additional suppressors of GAL-induced BRCA1 lethality. Many of these suppressors are functionally and temporally linked in mRNA metabolic processes that include transcriptional elongation, mRNA export and decay. Focusing on the process of elongation, we demonstrate in human cell extracts that BRCA1 can be co-immunoprecipitated with the human ortholog of Spt5p and hyperphosphorylated RNAPII. We show that BRCA1 interacts with the *C t*erminal *d*omain (CTD) of *RNA p*olymerase II (RNAPII) when the CTD is phosphorylated in a pattern specified by the CTDK-I kinase (CTK), to mediate *p*hospho-*CTD* (P-CTD) cleavage. This P-CTD cleavage is not induced by cancer-associated mutations in the BRCT domain (C-terminal domain of BRCA1) and is suppressed by genomic deletion of the RNAPII *de*gradation *f*actor *DEF1* and *CTK1,* the CTD kinase that results in phosphorylation of serine 2 and serine 5. We further show that UV-irradiation results in a proteasome dependent accumulation of the P-CTD cleavage product in yeast and human breast cancer cells wild type for BRCA1 but not in a BRCA1 mutant cell line. Ectopic expression of the wild type BRCA1 protein in this mutant line restores damage dependent P-CTD cleavage indicating a direct mechanistic link between BRCA1 and this newly discovered RNAPII processing event. From this and other supportive data, we propose a model in which BRCA1-induced RNAPII P-CTD cleavage and degradation is a key component of a highly conserved, checkpoint/surveillance pathway that detects DNA damage within actively transcribing genes.

## Results

### A genome-wide screen for suppressors of BRCA1-induced lethality in yeast identifies genes required for mRNA elongation, export and decay

**Figure 1:**
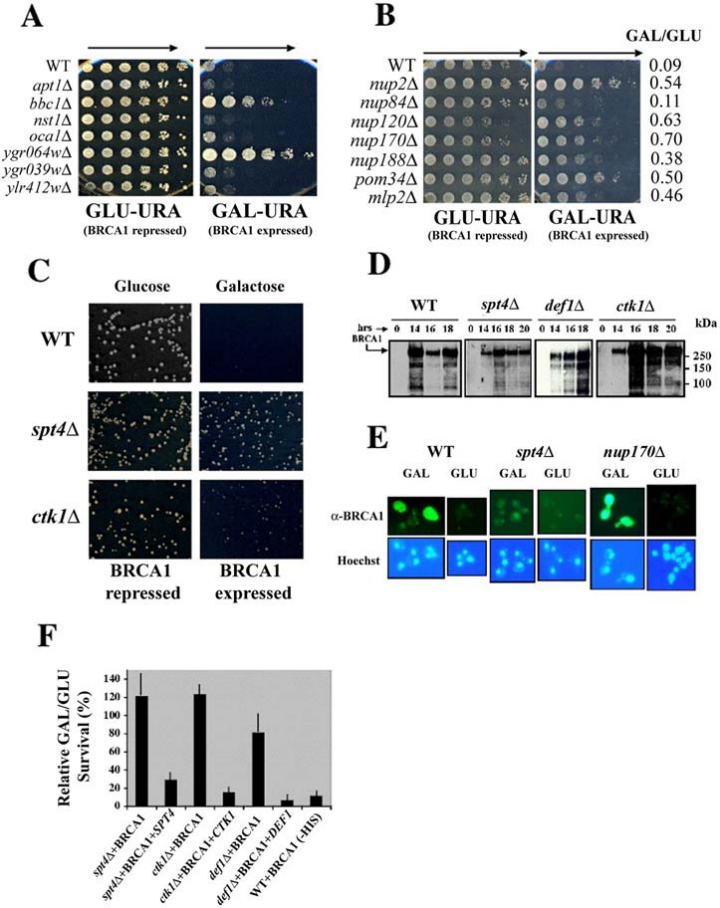
Identification and characterization of genetic suppressors of BRCA1-induced lethality in yeast. (A) Suppression of BRCA1-induced lethality following transformation of isogenic deletion strains with BRCA1 plasmid. BRCA1 suppressor strains were initially identified by sequencing rapid growing strains arising on GAL-URA plates following transformation of the diploid deletion strain pool with the *GAL::*BRCA1 expression plasmid. Putative BRCA1 suppressor strains identified from the pool were selected from the arrayed deletion strain library and transformed with the BRCA1 expression plasmid, grown to stationary phase in GLU and 2 ul aliquots were replica spotted onto GLU and GAL plates from serial 5-fold dilutions in water (arrows indicate direction of decreasing cell concentration). Typical results are depicted for seven strains. Five deletion strains (*apt1Δ, nst1Δ, oca1Δ, ygr039WΔ* and *ylr412WΔ*) failed to suppress BRCA1 lethality and were omitted from further study. Two deletion strains, *bbc1Δ* and *ygr064WΔ* (which deletes the N terminus of *SPT4*) were confirmed to suppress BRCA1-induced lethality. (B) Deletion strains defective in mRNA export suppress BRCA1-induced lethality. Deletions of the nuclear pore associated genes *NUP2, POM34* and *MLP2* (from deletion pool) and deletions of the IR resistance genes *NUP120, NUP170, NUP188* as well as *NUP133* (not shown, Table S1) suppress BRCA1 lethality. Relative plating efficiencies for colony formation on GAL *vs* GLU (GAL/GLU) are from Table S1. With the exception of *nup84Δ*, all IR sensitive nuclear pore defective strains significantly suppressed BRCA1-induced lethality. (C) Deletion of the transcriptional elongation genes *SPT4* or *CTK1* completely suppress BRCA1-induced lethality. Reduced colony forming ability on GAL (Galactose) *vs* GLU (Glucose) plates is completely suppressed by deletion of *SPT4* or *CTK1* when compared to WT strains containing the BRCA1 plasmid. Plates depicted are following 3 (WT and *spt4Δ* strains) and 4 days (*ctk1Δ*) incubation at 30°C. (D) Suppressor strains defective in transcription elongation express BRCA1. WT, *spt4Δ, def1Δ* and *ctk1Δ* strains containing the BRCA1 expression plasmid were induced in liquid GAL for the indicated times (hrs). Western blot has been probed with anti-BRCA1 antibody MS110. Full-length BRCA1 has an apparent MW of 220–250 kDa or larger (arrow). (E) Nuclear exclusion of BRCA1 is not responsible for suppression of BRCA1-induced lethality. *In situ* immunofluorescence of BRCA1 was determined in uninduced yeast cells (GLU) or induced for BRCA1 expression for 6 hours (GAL). Hoechst staining was used to position the nucleus. No partitioning defects were observed for BRCA1 in other nuclear pore mutants (*nup2Δ, nup120Δ, nup133Δ, nup188Δ, pom34Δ* or *mlp2Δ*; data not shown) that suppressed BRCA1 lethality. In all cells examined, BRCA1 was localized to both the nucleus and cytoplasm. Bar is 10 um. (F) Suppression of BRCA1-induced lethality is not due to second site suppressors. Diploid deletion strains (*spt4Δ, ctk1Δ* and *def1Δ*) containing the *GAL::*BRCA1 expression plasmid were “covered” with the corresponding gene expressed from a second selectable plasmid and assayed for the reinstatement of BRCA1 lethality by determining the relative plating efficiencies of colony forming ability on GAL *vs* GLU with selection for both plasmids. WT+BRCA1 (-HIS) is the relative survival of colony forming ability for the WT strain containing the *GAL::*BRCA1-*HIS3* expression plasmid on GAL-HIS *vs* GLU-HIS medium. Error bars are +/− 1 SD about the mean and each bar represents 3-6 experiments.

Although a BRCA1 ortholog is not found in the yeast *S. cerevisiae*, expression of BRCA1 in yeast results in slow growth and lethality. We reasoned that an interaction of BRCA1 with a conserved yeast protein(s) interferes with a basic cellular function essential for yeast survival and that these proteins would be intimately involved in the maintenance of genomic integrity. By screening for rapid growth on GAL-URA (GAL) medium immediately following transformation of the *BRCA1* expression plasmid into a pool of genetically tagged deletion strains, we initially identified a total of 48 mutant strains that suppressed BRCA1 lethality. Typically upon screening, the WT strain containing the BRCA1 plasmid failed to grow on GAL beyond the first or second dilution (Fig. 1A,B) and the relative colony forming survival fraction was 0.098±0.089 (*i.e.,* 90.2% of WT cells fail to survive BRCA1 induction; n = 33±1 SE; Fig. 1C). Of the 48 deletion strains that were initially identified to suppress BRCA1, 20 (42%) were subsequently confirmed to suppress lethality greater than 2 fold over WT in an independently transformed yeast deletion isolate (Table S1).

As a secondary screen, we also examined the 203 deletion strains that we previously found to be radiation sensitive [11] [12]. Thirteen of these were found to suppress BRCA1 lethality (Table S1). These included *dhh1Δ* and *ccr4Δ*, previously characterized as suppressors [8], *def1Δ* which is involved in RNAPII degradation following DNA damage [13], a number of nuclear pore proteins required for mRNA transport, as well as two open reading frames (ORFs; *yml009c-a* and *yml009w-b*) that overlap the 3′ end of *SPT5* to truncate the protein. We collectively identified mutants with defects in highly conserved cellular processes that primarily affect mRNA metabolism. Specifically, these processes include transcription elongation (*DEF1, SPT4, SPT5, SUB1*), mRNA export (*MLP2, ASM4, NUP2, NUP120, NUP133, NUP170, NUP188, POM34;* Fig. 1B) and mRNA decay at cytoplasmic processing bodies (*DHH1, CCR4*). Using these screening approaches, we identified a total of 36 deletion strains that suppressed BRCA1 lethality (Table S1).

Extensive genome-wide proteomic and genetic interaction networks have been characterized in yeast. We utilized these interaction networks (listed at SGD) to determine whether genes or gene products that genetically or physically interact with experimentally identified suppressors of BRCA1 (Table S1) could identify additional deletion mutations that suppress BRCA1 lethality. Using known network interactions, we identified a further 9 deletions including *mlp1Δ* and *nup53Δ* as suppressors of BRCA1. Moreover, genetic interactions of BRCA1 with RNAPII elongation components predicted *ctk1Δ* and *srb10Δ* (*ssn3Δ)* as suppressors of BRCA1-induced lethality due to their impact on RNAPII C-terminal domain (CTD) phosphorylation (described below). Thus a total of 45 suppressors of BRCA1 lethality were identified (Table S1).

### Suppression of lethality is not due to reduced BRCA1 expression, nuclear exclusion or second site suppressors

Since suppressors of BRCA1 lethality could have an impact upon mRNA expression and export or nuclear import of the BRCA1 protein, we analyzed representative strains for BRCA1 expression and subcellular localization. Following a standard whole cell extraction protocol using ice-cold NP40 buffer, BRCA1 was extensively degraded even in the presence of protease inhibitors (data not shown). To alleviate this problem we adopted an extraction protocol in 95% ethanol that rapidly fixes the cells and greatly reduced BRCA1 degradation (see Materials and Methods). GAL-induced expression of BRCA1 protein was unaffected by deletion of the major transcriptional regulators that suppress lethality when compared to WT (Fig. 1D). Furthermore, intranuclear localization of GAL-induced BRCA1 was unaffected in suppressor mutants that regulate transcription (*spt4Δ*) or nuclear pore functions (*nup170Δ*) as determined by immunofluorescent detection (IF, Fig. 1E). Further, we “covered” three of the major suppressor deletion strains by expressing the *SPT4*, *CTK1* and *DEF1* genes. In each case, BRCA1 lethality was restored on GAL indicating that second site mutations were not responsible for the suppressor phenotype (Fig. 1F). Thus suppression of BRCA1 lethality appears to result from the identified gene deletions and absence of the corresponding gene products that functionally mediate the lethal BRCA1 response.

### Recombination and TCR repair are dispensable for BRCA1-induced lethality

In human cells, BRCA1 has been shown to interact with components of the recombinational repair apparatus (Rad51p) as well as components of the non-homologous end-joining pathway which are all highly conserved in yeast and required for DSB repair (reviewed in [14] [15]). We therefore examined strains individually deleted for members of the RAD52 epistasis group to determine if lethality was suppressed following GAL induced expression of BRCA1. Only some repair defective strains (*i.e. rad50Δ, rad51Δ, rad52Δ, rad55Δ* and *xrs2Δ*) demonstrated a modest suppression of BRCA1-induced lethality (Table S1). No suppression of BRCA1-induced lethality was observed for *rad54Δ, rad57Δ* or *rdh54Δ* strains (data not shown). Since *DEF1* [16] and *SPT4* [17] have been linked to transcription-coupled repair (TCR), we also examined a number of strains (*rad7Δ, rad16Δ, rad23Δ, rad26Δ, rad28Δ, met18Δ, rpb4Δ* and *rpb9Δ*) deleted for genes that have been implicated in TCR to determine if any were required for BRCA1-induced lethality. Following deletion of components of the yeast TCR apparatus, little (*RAD16, MET18*, *RPB4*; Table S1) or no (*RAD7*, *RAD23, RAD26, RAD28*, *RPB9*; data not shown) suppression of BRCA1-induced lethality was observed. These results suggest that recombination, non-homologous end-joining and TCR-mediated repair pathways are not responsible for the majority of the BRCA1-induced lethality in yeast.

### The DSIF complex is required for BRCA1-induced cell cycle arrest and the maintenance of genomic integrity following DNA damage

**Figure 2:**
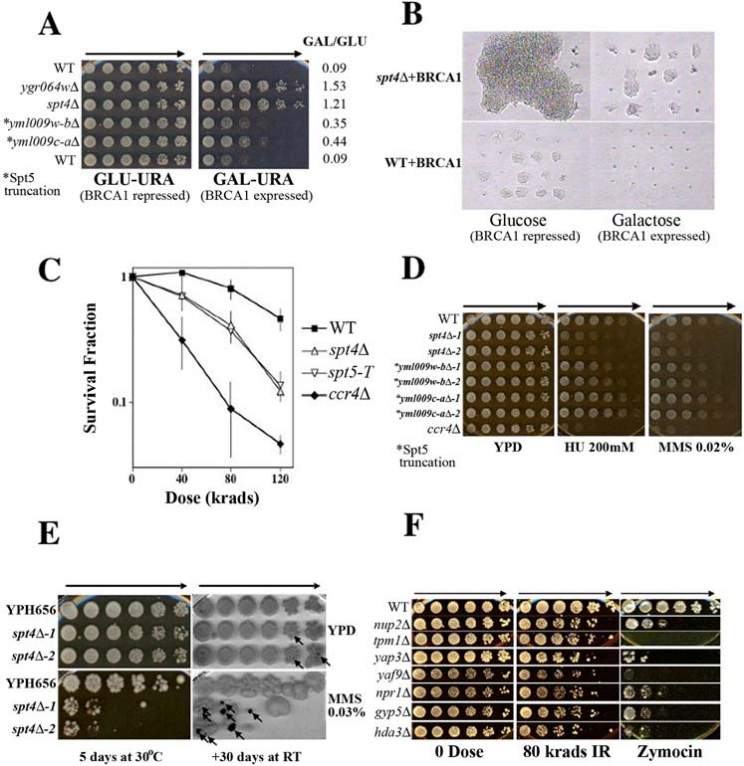
BRCA1 suppressor strains are sensitive to DNA damaging agents. (A) DSIF defective strains suppress BRCA1-induced lethality. Deletion of the *ygr064WΔ* ORF deletes the N terminus of *SPT4*. Full-length deletion of *SPT4* also completely suppressed BRCA1 lethality. Spt4p physically interacts with Spt5p in the DSIF complex to confer transcription elongation functions. *SPT5* is an essential gene, however the relative survival of two strains (*yml009W-bΔ* and *yml009c-aΔ*) containing non-lethal C terminal truncations of *SPT5* was enhanced when compared to WT following BRCA1 expression. Relative colony forming ability for these strains on GAL *vs* GLU (GAL/GLU) has been indicated. (B) Deletion of *SPT4* suppresses prolonged G1 arrest following BRCA1 expression in yeast. Individual unbudded (G1) cells were positioned using a micromanipulator into 4×5 cell grid patterns on GLU-URA and GAL-URA plates. None of the WT cells expressing BRCA1 (on GAL) were able to progress beyond the single cell (G1) stage of the cell cycle by 24 hours. However, deletion of *SPT4* allowed most of the cells (13/20) to progress into microcolonies following BRCA1 expression. When BRCA1 was repressed (on GLU), most of the WT and the *spt4Δ* cells progressed beyond the G1 stage of the cell cycle to form microcolonies. (C) DSIF defective yeast strains are sensitive to γ-irradiation. WT, *spt4Δ* and *spt5* mutant (*yml009w-bΔ*) strains were grown to stationary phase and exposed to γ-irradiation as previously described [12]. WT survival data was pooled with previous data (n = 10 experiments) to generate the γ-ray WT survival curve depicted above. Survival fractions for the *spt4Δ* and *spt5* strains are averaged from 3-4 experiments. Previously published survival data for the *ccr4Δ* strain [12] has been included for comparison. Error bars are +/− 1 SE about the mean. (D) Diploid *spt4Δ* strains are sensitive to the S phase specific DNA damaging agents HU and MMS. WT, *spt4Δ, spt5* truncated (*yml009w-bΔ; yml009c-aΔ*) and *ccr4Δ* strains were exposed as described in Methods. Arrows indicate the direction of decreasing cell concentrations. The *ccr4Δ* strain has been included as a positive control. (E) *SPT4* maintains chromosome stability following DNA damage. The WT (YPH656) and isogenic *spt4Δ* YPH656 derived strain contain a stable YAC that carries the *SUP11* gene that allows read through of the ochre mutation within *ade2-101*. Strains that retain the YAC remain white while loss of the YAC results in a red colony. Strains were grown and diluted as described in Panel D. Two independent *spt4Δ* isolates demonstrate MMS hypersensitivity and enhanced MMS-induced chromosome loss as indicated by numerous (n = 10) red Ade- colonies (arrows) when compared to the WT parental YPH656 strain (no red colonies). Spontaneous YAC loss was observed in untreated *spt4Δ* strains. (F) BRCA1 suppressor strains are sensitive to γ-irradiation and hypersensitive to zymocin. Strains were grown and diluted as described in Panel D. Diluted cells were replica spotted onto YPD and either left unirradiated (0 Dose), exposed to 80 krads of γ-irradiation or plated to YPD containing zymocin toxin secreted from *K. lactis* as previously described [12].

Spt4p and Spt5p physically interact in the DSIF (5,6-*d*ichloro-1-beta-D-ribofuranosylbenzimidazole *s*ensitivity *i*nducing *f*actor) complex with RNAPII to affect transcription elongation in both yeast [18] and human cells [19]. Although *SPT5* is an essential gene in yeast, two overlapping ORFs from the deletion collection truncate the carboxy terminal end of *SPT5* yet retain viability (*yml009w-bΔ, yml009c-aΔ,* Table S1). Furthermore, *ygr064wΔ* deletes the N terminus of Spt4p. These deletion strains all suppressed BRCA1 lethality (Fig. 2A) and similar to isogenic *dhh1Δ* and *ccr4Δ* strains, which have a checkpoint adaptation function [8] [12], deletion of *SPT4* suppressed the prolonged G1 arrest induced by expression of BRCA1 (Fig. 2B). DSIF defective yeast strains also exhibit IR sensitivity (Fig. 2C) previously described for the *SPT5* truncations [11]. Furthermore DSIF defects resulted in enhanced sensitivity to the S phase specific damaging agents HU and MMS (Fig. 2D,E). Moreover, the *spt4Δ* allele resulted in enhanced chromosome instability, both spontaneously and following MMS exposure, when transferred into a strain that detects enhanced loss of a stable YAC (yeast artificial chromosome; Fig. 2E). These data indicate a shared role for the DSIF complex in DNA damage tolerance, maintenance of genomic stability and suppression of BRCA1-induced lethality in yeast. Taken together, these results suggest that BRCA1 subverts a conserved DNA damage response pathway in yeast in which DSIF plays a critical role.

### Other BRCA1-interactive targets mediate resistance to DNA damaging agents

Many of the newly identified suppressors of BRCA1-induced lethality have not been previously associated with DNA damage sensitivity. Testing these strains, we found that most of the suppressor strains (11/17) demonstrated enhanced IR sensitivity (∼5–10 fold) when compared to WT. Further, all of these strains were hypersensitive to the toxin zymocin (Fig. 2F) that arrests cells in the G1 phase of the cell cycle and affects a number of RNA metabolic processes including transcription elongation [12]. These results lend additional support for the biological relevance of this screen since we identified proteins involved in the maintenance of genomic integrity following DNA damage, a BRCA1-dependent process in human cells.

### BRCA1-induced lethality in yeast is mediated by genome instability and plasmid loss

**Figure 3:**
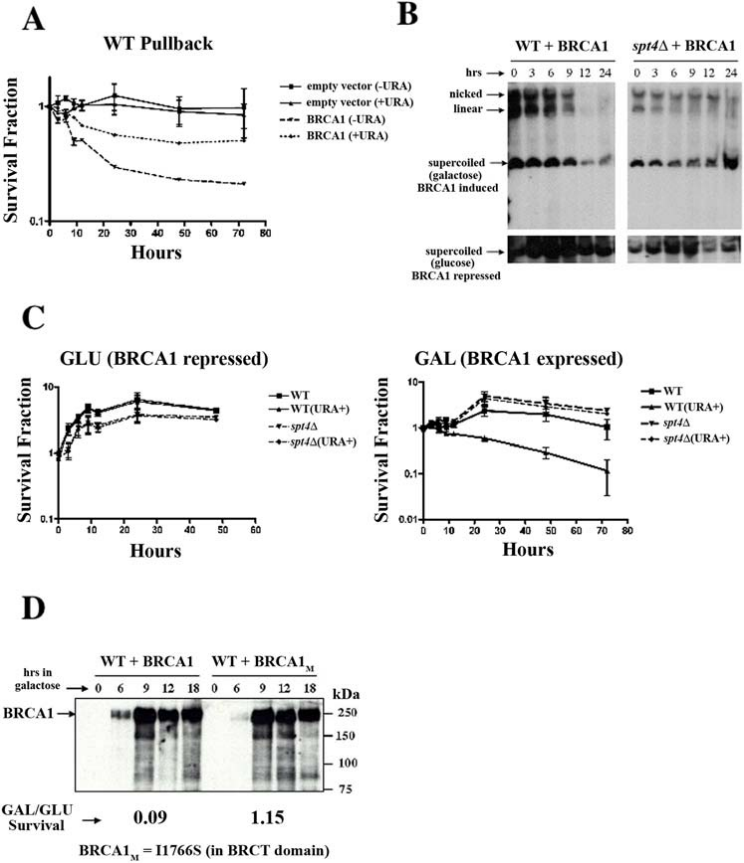
BRCA1-induced lethality involves plasmid loss and requires a functional BRCA1-BRCT domain. (A) “Pullback” of WT yeast containing either the BRCA1 expression plasmid or empty vector control. To induce BRCA1, dilutions of plasmid bearing cells were plated into cooled liquid GAL agar containing medium with (+URA) or without (-URA) uracil selection for the plasmid marker (*URA3*) as previously described [54]. At the indicated times, BRCA1 expression was repressed by overlaying with GLU+URA or GLU-URA agar. For some data points, error bars (+/− 1 SD) are contained within the symbol. Enhanced lethality under selective conditions indicates plasmid loss is a significant component of BRCA1-induced lethality. (B) Deletion of *SPT4* suppresses the physical loss of the BRCA1 expression plasmid. Stationary cultures of WT and *spt4Δ* strains containing the BRCA1 plasmid were “split” into twice the volume of GAL or GLU to express or repress BRCA1 respectively. Expression of BRCA1 in WT cells (in GAL) resulted in a time dependent increase in plasmid loss as determined by Southern analysis. Cells deleted for *SPT4* retain the BRCA1 plasmid following BRCA1 expression. When BRCA1 was repressed, plasmid was physically retained in both WT and *spt4Δ* strains. (C) *SPT4* deletion suppresses plasmid loss following BRCA1 expression. The viability of cells from the experiment described in Panel B was determined by plating aliquots at the indicated times onto YPD agar medium and genetically determining plasmid loss by replica plating the resulting colonies onto GLU-URA plates to detect the presence or absence of the plasmid *URA3* marker (URA+). The relative survival of colony forming ability following repression (in GLU; left panel, two experiments) or induction of BRCA1 (in GAL; right panel, four experiments) in liquid media is shown. A significant loss of the plasmid marker can be observed in the WT but not in the *spt4Δ* strain following BRCA1 expression. No significant loss of the BRCA1 plasmid can be seen for either strain when the cells were grown in GLU. (D) Breast cancer associated mutations within the BRCT domain of BRCA1 suppress lethality in yeast. A single base pair was mutated within the BRCA1 expression plasmid (I1766S). The relative survival of colony forming ability on GAL *vs* GLU (GAL/GLU survival, four experiments) was determined as described for Fig. 1B. Complete suppression of BRCA1-induced lethality in yeast was also observed following deletion of the C-terminal 10 amino acids of BRCA1 that also disrupts BRCT domain function (data not shown).

Loss of BRCA1 function in human cells results in genome instability. We therefore investigated whether expression of BRCA1 in yeast was also genome-destabilizing and could account for some or all of the lethality observed in yeast. We first examined whether lethality was dependent on plasmid selection following expression of BRCA1 (Fig. 3A). In order to determine the kinetics of BRCA1-induced plasmid loss and subsequent lethality, we performed a “pullback” experiment in which WT cells containing the BRCA1 plasmid or empty vector were plated within GAL-URA or GAL+URA medium to induce BRCA1 expression. At increasing time intervals, we overlaid the GAL plates with either GLU-URA or GLU+URA medium to repress BRCA1 expression. When plasmid selection (-URA) was maintained throughout the experiment so that only plasmid retaining cells would survive, a rapid time dependent decrease in survival was observed. Following plating under non-selective conditions (+URA) survival was still significantly reduced as compared to WT cells containing empty vector, however survival was increased as compared to that under selective conditions indicating that loss of the plasmid selectable marker comprises only part of the observed BRCA1-induced lethality (Fig. 3A). We also directly analyzed plasmid loss in both WT and *spt4Δ* by Southern blotting (Fig. 3B). Supporting the growth result, dramatic plasmid loss is evident in WT. In contrast, the efficient suppressor of lethality, *spt4Δ* retained plasmid throughout the experiment. We also demonstrated plasmid retention in *spt4Δ* using a similar pullback approach following BRCA1 expression in liquid medium (Fig. 3C). Thus plasmid and chromosomal instability appears to play a key role in BRCA1-induced lethality in yeast.

### BRCA1-induced lethality in yeast requires the BRCT domain

In yeast, slow growth following expression of BRCA1 [7] was shown to be associated with the BRCT domain [20]. We separately introduced two breast cancer associated BRCT mutations into the yeast *BRCA1* expression plasmid. In one construct, we introduced a single nucleotide change that resulted in a BRCT amino acid substitution (I1766S). For the second mutation, we introduced a premature stop codon that truncated the BRCA1 protein by deleting the last 10 C-terminal amino acids. Following GAL induction, both mutant proteins were expressed in amounts similar to that observed for the full-length WT BRCA1. Neither of these mutant BRCA1 proteins reduced yeast survival following expression (Fig. 3D; data not shown). To extend this observation, we cloned the final 333 amino acids of BRCA1 containing the tandem BRCT domains into the yeast high copy expression plasmid pDEST52. Similar to full-length BRCA1, expression of the BRCT construct in WT yeast resulted in significant but slightly reduced lethality (relative survival fraction = 0.21±0.06; ±1 SE). Thus, the majority of the lethality observed in yeast following expression of full-length BRCA1 appears to be mediated through the BRCT domain.

### CTDK-I (*CTK1* kinase) is required for BRCA1-induced lethality in yeast

A critical and unique feature of elongating RNAPII is its hyperphosphorylated and highly conserved CTD domain. The identity of several suppressor strains as well as the ability of BRCA1 to modulate RNAPII CTD phosphorylation [21] suggested a connection between BRCA1 and transcription elongation by RNAPII. We therefore tested whether deleting genes in subunits of three different CTD kinases could suppress BRCA1 lethality. We found that deleting *SRB10* or *BUR2*, had only a modest impact on the suppression of BRCA1 lethality. Remarkably, deleting *CTK1* completely suppressed BRCA1 lethality (Table S1, Fig. 1C). This result argues that BRCA1 lethality requires *CTK1*-dependent phosphorylation of the RNAPII CTD, and suggests that the molecular mechanism of BRCA1 lethality requires the pattern of CTD phosphorylation specified by CTDK-I [22] [23].

### BRCA1 genetically interacts with the CTD of RNAPII

**Figure 4:**
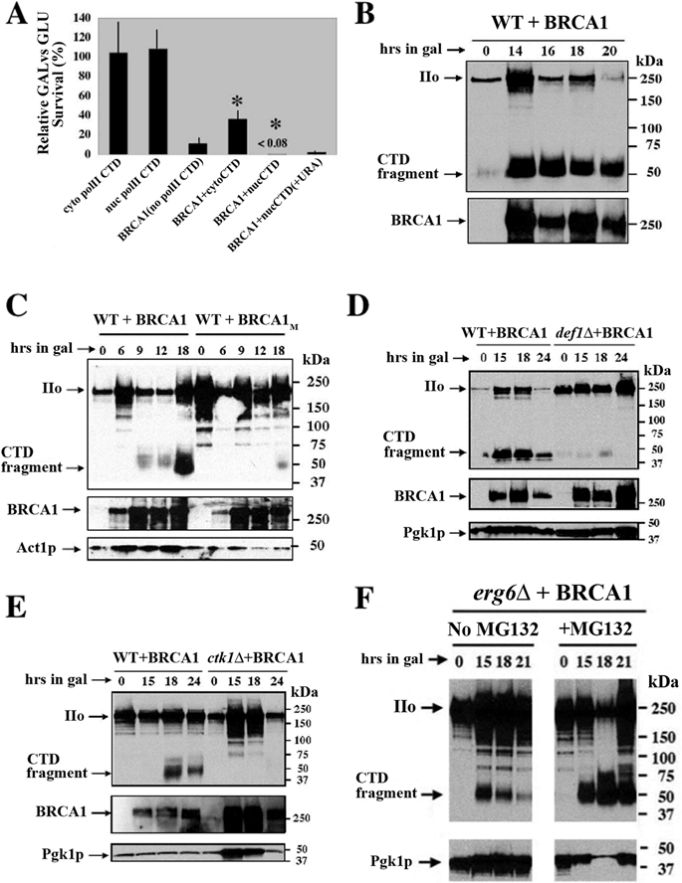
BRCA1 interacts with the phosphorylated RNAPII CTD to induce cleavage and proteasome-mediated degradation. (A) BRCA1 genetically interacts with the RNAPII CTD in WT yeast. WT yeast cells were transformed with plasmids that express the RNAPII CTD in the nucleus (nuc-polII CTD) or cytoplasm (cyto-polII CTD) due to the presence or absence of a NLS signal sequence. A second selectable (*HIS3*) *GAL::*BRCA1 expression vector was subsequently established in these strains by transformation. Relative plating efficiencies on GAL *vs* GLU was determined as described in Fig. 1B with plasmid selection. Co-expression of the BRCA1 and the cyto-CTD construct significantly (*) suppressed BRCA1-induced lethality, whereas co-expression of BRCA1 and the nuc-CTD construct significantly (*) augmented lethality. The relative survival fraction for cells containing both the BRCA1 and nuc-CTD plasmids was less than (<) 0.08 since no colonies appeared on the GAL-HIS-URA plates. This augmented lethality could be partially “relieved” by plating cells to non-selective medium where retention of the nuc-CTD construct was not required for viability (in medium containing uracil; +URA). This indicated that nuc-CTD plasmid loss occurred *in trans* following BRCA1 expression. Error bars are the average of 4-6 experiments (+/− 1 SD). (B) BRCA1 expression in WT yeast induces cleavage of the RNAPII CTD. Following BRCA1 expression in WT cells, Western blots were probed with anti-2,5P CTD antibody (anti-2,5P, upper panel), stripped and re-probed with anti-BRCA1 antibody (lower panel). The positions of the intact phosphorylated RNAPII (IIo) and a novel BRCA1-induced 50 kDa phospho-CTD (P-CTD) fragment have been indicated. (C) WT yeast that express BRCA1 with a BRCT domain defect fail to induce P-CTD cleavage. WT cells containing BRCA1, BRCT mutated (I1766S) or empty plasmid expression vectors were induced for BRCA1 expression in GAL and extracted at the times indicated. Western blots utilized anti-2,5P CTD (upper panel) and anti-BRCA1 (middle panel) antibodies as probes. Anti-Act1p antibody (bottom panel) served as a loading control. P-CTD cleavage in cells containing the empty vector was identical to that seen for the BRCT mutant expression plasmid (data not shown). (D) Deletion of *DEF1* inhibits BRCA1-induced P-CTD cleavage. WT and *def1Δ* strains containing the BRCA1 plasmid were induced for BRCA1 expression and extracted at the indicated times. Anti-2,5P CTD (upper panel) and anti-BRCA1 (middle panel) antibodies were utilized as Western blot probes. Pgk1p served as a loading control unaffected by the switch from GLU to GAL (bottom panel). The P-CTD fragment is greatly diminished in the *def1Δ* strain when compared to an equivalently loaded WT induced for BRCA1 expression. (E) Deletion of *CTK1* inhibits BRCA1-induced P-CTD cleavage. WT and *ctk1Δ* strains containing the BRCA1 plasmid were induced for BRCA1 expression and extracted at the indicated times. Cells were extracted, proteins separated and Western blotted as described in C above. The P-CTD fragment is undetectable in the *ctk1Δ* strain using either anti-2,5P or H14 anti-5 P antibody (data not shown). (F) MG132 inhibits degradation of the BRCA1-induced P-CTD fragment in yeast. Deletion of *ERG6* facilitates uptake of the proteasome inhibitor MG132 in yeast. We therefore established the BRCA1 expression plasmid within the *erg6Δ* strain and induced BRCA1 expression in the absence (left panels) and presence of MG132 (right panels). Persistence of the P-CTD fragment is enhanced by MG132 treatment indicating that the P-CTD fragment is targeted for proteasome-mediated degradation.

Since the DSIF complex and the Ctk1p suppressor kinase interact with the elongating form of RNAPII and the regulatory CTD domain of Rpb1p plays a key role in transcription elongation [24], we investigated whether overexpression of the yeast RNAPII CTD could influence BRCA1 lethality. To do this we constructed a C-terminal fusion of the yeast RNAPII CTD to β-galactosidase with and without a nuclear localization signal (NLS). These constructs were inserted into a selectable *GAL1* expression vector and following expression of these fusion protein constructs, the β-galactosidase-CTD fusion was found predominantly in the cytoplasm (cyto-CTD; no NLS) or in the nucleus (nuc-CTD; with NLS) as determined by *in situ* IF (data not shown). In WT yeast that concomitantly express BRCA1 from a second selectable plasmid, expression of the cyto-CTD significantly suppressed BRCA1-induced lethality whereas expression of the nuc-CTD significantly augmented the lethality of BRCA1 (Fig. 4A). Part of this augmented BRCA1-induced lethality appears to be directed towards enhanced nuc-CTD plasmid instability as addition of uracil (*URA3* is the selectable marker for the CTD expression plasmids) to the GAL plates decreased the lethal effects of co-expressing BRCA1 and the RNAP II CTD in the nucleus. Thus RNAPII CTD co-expression significantly altered the degree of BRCA1 lethality and indicated a genetic interaction between BRCA1 and the expressed RNAPII CTD.

### BRCA1 expression in yeast results in cleavage of the RNA polymerase II P-CTD

Given the genetic connections between BRCA1 lethality and the Rpb1p CTD, we examined the status of RNAPII following BRCA1 expression in yeast, employing a polyclonal antibody raised and purified against a synthetic CTD repeat peptide phosphorylated on every Ser2 and Ser5 residue (anti-2,5P), the pattern of phosphorylation preferentially generated by CTDK-I (*CTK1* kinase, [22] [25]). Unexpectedly, the predominant band observed in Western blots of extracts from cells expressing BRCA1 was not the intact, hyperphosphorylated Rpb1p subunit (IIo), but a band with an electrophoretic mobility of 50 kDa (Fig. 4B). This 50 kDa species appears to represent the P-CTD, cleaved from the rest of the Rpb1p subunit (the N-terminal fragment would not be detected by the anti-2,5P antibody). Its mobility is what might be expected for a segment of Rpb1p containing the entire hyperphosphorylated CTD plus an additional ∼100 amino acids N-terminal to the CTD. The appearance of this P-CTD fragment depends on the expression of WT BRCA1 (Fig. 4B) since it was observed in only very limited amount after expression of the two disease associated BRCA1 mutants or after galactose induction of cells containing empty vector (Fig. 4C; data not shown). BRCA1-induced accumulation of the P-CTD fragment also was found to be dependent on the *DEF1* gene (Fig. 4D) whose product has been implicated in ubiquitin-mediated proteasomal degradation of RNAPII stalled following UV damage [13]. Furthermore, BRCA1-induced accumulation of the P-CTD fragment was similarly reduced in the *ctk1Δ* strain even though BRCA1 was expressed at levels comparable to that observed in the isogenic WT strain (Fig. 4E). BRCA1-induced accumulation of the P-CTD fragment was enhanced by concomitant treatment with the proteasome inhibitor MG132 in the permeable *erg6Δ* strain suggesting that the P-CTD was subject to proteasome-mediated degradation following cleavage (Fig. 4F). To confirm the identity of the 50 kDa species, we were able to detect this band after BRCA1 expression in strains that express an Rpb1p allele with a C-terminal His_10_ tag or a reduced number of heptapeptide repeat units in the CTD (Figure S1). Taken together, these results indicate that expression of BRCA1 with an intact BRCT domain in yeast leads to the appearance of a novel P-CTD fragment, cleaved from a form of RNAPII whose phosphorylation state is dependent on CTDK-I. Subsequent stability of this fragment is influenced by the proteasome system.

### BRCA1 interacts with the DSIF complex in yeast and human cells

**Figure 5:**
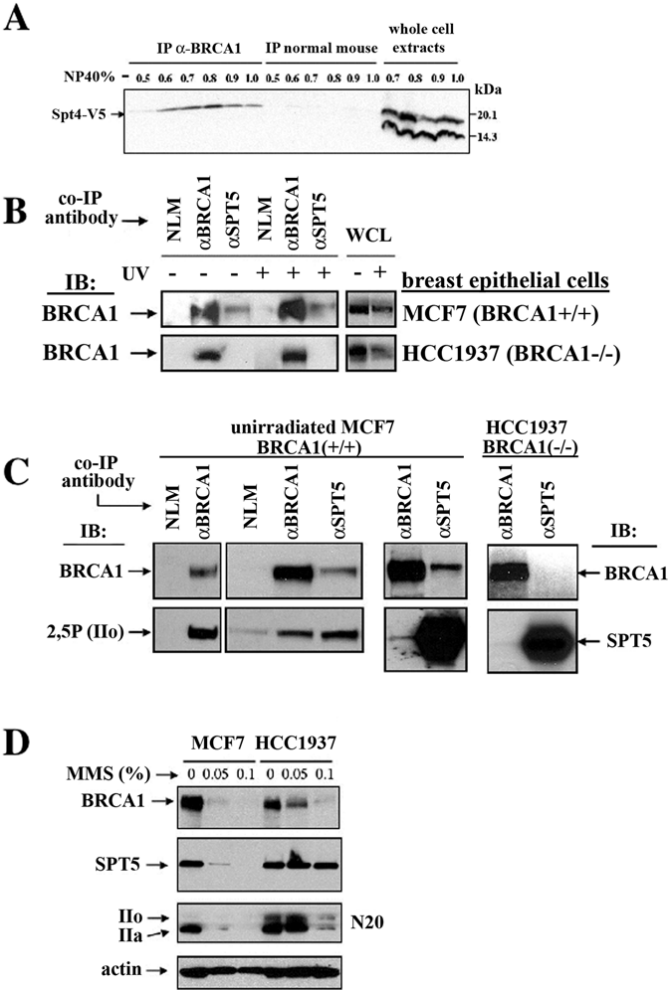
BRCA1-DSIF complexes are rapidly degraded. (A) Spt4p physically interacts with BRCA1 in yeast. WT cells bearing the *GAL::*BRCA1 and *GAL::*Spt4-V5-*LEU2* expression plasmids were induced for co-expression of BRCA1 and Spt4-V5 (16 hrs). Whole cell lysates (WCL) were prepared in various concentrations of NP40 and either loaded directly (200 ug/lane) or subjected to co-IP reactions overnight using anti-BRCA1 antibody or normal mouse IgG. Proteins were separated by SDS-PAGE and subjected to Western blotting using anti-V5 antibody as a probe. Spt4p is detected as a double band in WCL of WT cells migrating between 14 and 20 kDa. This doublet Spt4p band was also detected endogenously using anti-Spt4p antibody (sc-26353, Santa Cruz) as a probe (data not shown). The upper Spt4-V5 band was observed to interact maximally with BRCA1 at an NP40 concentration of 0.8%. BRCA1 similarly interacted with endogenous Spt4p when anti-Spt4p antibody (sc-26353, Santa Cruz) was used as the IP antibody (data not shown). (B) Interaction of BRCA1 with SPT5 is dependent on the BRCA1-BRCT domain in human cells. MCF7 and HCC1937 cells were either left untreated or exposed to UV (20 J/m^2^) for 6hr in the presence of the proteasome inhibitor MG132 (50 uM). WCL were prepared and subjected to co-IP analysis using normal mouse IgG (NLM), anti-BRCA1 or anti-SPT5 mouse monoclonal antibody as the IP reagent. Western immunoblot (IB) analysis utilized anti-BRCA1 as a probe. The interaction of SPT5 with BRCA1 is observed in both unirradiated and UV-irradiated MCF7 cells. Interaction between BRCA1, and SPT5 is abolished in HCC1937 cells. At the time of irradiation, cells were treated with the proteasome inhibitor MG132 (50 uM) to inhibit rapid damage-induced degradation of the BRCA1-SPT5 complex. In the absence of proteasome inhibition, no BRCA1-SPT5 interaction was seen following UV irradiation (data not shown). (C) BRCA1 interacts with SPT5 and RNA polymerase II in human cells. Co-IP reactions using the indicated monoclonal antibodies and normal (non-immune) mouse IgG (NLM) were separated by SDS-PAGE. Western immunoblot (IB) analysis utilized, anti-BRCA1, anti-2,5P and anti-SPT5 as probes. Both BRCA1 and SPT5 interacted with the full-length hyperphosphorylated form of RNA polymerase II (IIo). SPT5 interaction with BRCA1 could be detected using either anti-BRCA1 or anti-SPT5 monoclonal antibodies as the immunoprecipitation reagent. Interaction between SPT5 and BRCA1 was abolished in HCC1937 cells that express a BRCT defective form of BRCA1. (D) Rapid MMS-induced degradation of BRCA1, SPT5 and RNAPII is BRCT domain dependent. MCF and HCC1937 cells were either left unexposed or exposed to the radiomimetic methyl methanesulfonate (MMS) for 2 hours at 37°C. Separated proteins were Western immunoblotted using anti-BRCA1, anti-SPT5, anti-RNAPII antibody N20 and anti-actin as probes. Whereas MMS treatment of MCF7 cells (BRCA1+/+) led to a dramatic degradation of BRCA1, SPT5 and RNAPII (Rpb1 subunit). MMS treatment of HCC1937 cells (BRCA1−/−) resulted in reduced and/or delayed degradation of the three proteins. These results suggest that the co-degradation may be a BRCT domain dependent process.

Suppression of BRCA1-induced lethality in *spt4Δ* and *spt5* yeast strains suggests that the DSIF complex may mediate interaction of BRCA1 with RNAPII. Therefore, we tested whether components of the DSIF complex physically interact with BRCA1 in yeast and human cells. Following expression of a V5 tagged Spt4p in yeast co-expressing BRCA1, we were able to co-IP BRCA1 and Spt4p (Fig. 5A). As our genetic screen in yeast was intended to identify novel BRCA1 interactive targets in mammalian cells, we turned our attention to a more physiologically relevant system. Using lysates from the MCF7 breast cancer cell line that harbors wild type BRCA1, we detected an interaction between endogenous SPT5 and BRCA1 by co-IP analysis. This interaction was present both in unirradiated cells (Fig. 5B,C) and in cells following 20 J/m^2^ of UV-irradiation (Fig. 5B). In HCC1937 cells harboring a hemizygous BRCA1 mutation (5382insC resulting in mistranslation starting at codon 1755 and premature termination at 1829), we found no detectable interaction between these two proteins, with or without DNA damage suggesting that the BRCA1 C-terminal domain is required. In MCF7 cells the interaction was detectable in both directions using either SPT5 or BRCA1 as a capture protein (Fig. 5C, right half). Finally, interactions between SPT5, BRCA1 and the phosphorylated full-length form of RPB1 (IIo) were also detectable in unirradiated MCF7 cell extracts (Fig. 5C, left half).

### MMS treatment induces rapid degradation of BRCA1, SPT5 and RPB1

BRCA1 appears to have ubiquitin ligase activity, with phosphorylated RPB1 acting as a substrate in a DNA damage responsive manner [26] [27] . Since SPT5 appears to be associated with both BRCA1 and the RNA polymerase, we asked whether its degradation was influenced by BRCA1 or DNA damage. Treatment of MCF7 and HCC1937 cells with the radiomimetic alkylating agent MMS for 2h induced a dramatic dose dependent decrease in steady-state SPT5 levels in the BRCA1 wild type MCF7 cells but not the mutant HCC1937 line (Fig. 5D). Loss of BRCA1 and RPB1 was also much more dramatic in MCF7 cells providing further evidence for a functional link between these proteins. Proteasome inhibition only partially prevented the damage-induced loss of all three proteins in MCF7 cells (data not shown). Dependence of damage-induced degradation of BRCA1, SPT5 and RPB1 on a functional BRCA1-BRCT domain suggests that this BRCA1-mediated degradation pathway may be critical for the suppression of tumorigenesis. Moreover, the BRCA1-mediated co-degradative loss of the abundant SPT5 protein along with RNA polymerase II suggests that the DSIF complex may have a key role in maintaining RNAPII stability prior to DNA damage.

### P-CTD cleavage is induced following DNA damage in yeast and human cells

**Figure 6:**
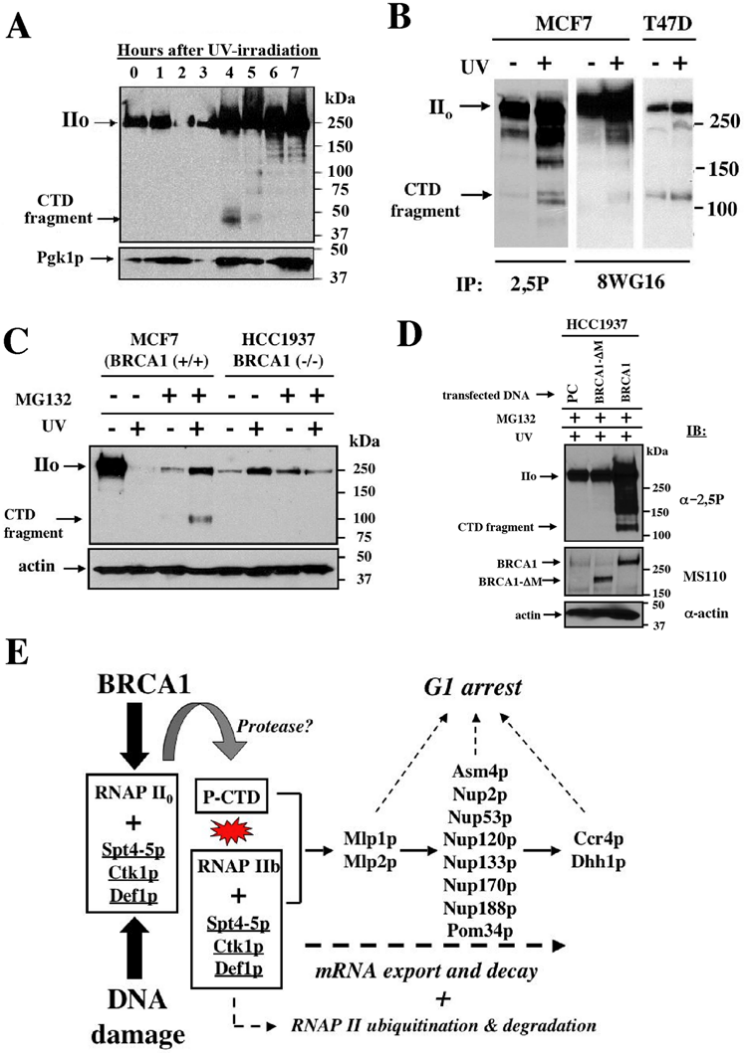
DNA damage induces RNAPII P-CTD cleavage in yeast and human cells. (A) UV damage in yeast induces RNAPII P-CTD cleavage. Stationary phase WT diploid yeast cells were irradiated with 120 J/m^2^ of UV-irradiation. UV induced a transient 50 kDa RNAPII P-CTD cleavage fragment at 4–5 hours following irradiation and outgrowth in fresh YPD medium. This fragment was similar in size to that observed following BRCA1 expression. The survival fraction for colony forming ability following UV irradiation was 0.70. P-CTD cleavage was not observed in unirradiated control cells (data not shown). (B) Antibodies specific for the RNAPII CTD immunoprecipitate a RNAPII P-CTD fragment following UV irradiation. WCL were prepared from unirradiated and UV-irradiated (20 J/m^2^) MCF7 and T47D cells 4 hours after irradiation in the presence of MG132 and subsequently immunoprecipitated with either 2,5P or 8WG16 anti-CTD antibodies. 8WG16 is specific for unphosphorylated and partially phosphorylated forms of Rpb1. Immunoprecipitates were separated on polyacrylamide gels and the P-CTD detected by Western blot using the 2,5P anti-CTD antibody as described above. UV damage enhanced the ability to detect the P-CTD fragment by immunoblotting following immunoprecipitation (IP) with either 2,5P or 8WG16 antibody. Although P-CTD fragment was detected in extracts from unirradiated cells, the ability to IP the P-CTD fragment was enhanced in extracts from UV-irradiated cells. Images depict proteins greater than 75 kDa. Cross-reacting heavy chain antibodies used in the IP reactions were detected at ∼50 kDa (data not shown). (C) UV-irradiation induces RNAPII P-CTD cleavage in human cells. Human cell lines MCF7 and HCC1937 were grown to 70–80% confluence and either left unirradiated or irradiated with UV (20 J/m^2^). Unirradiated (−) and UV-irradiated (+) cells were subsequently incubated at 37°C in growth medium with (+) or without (−) the proteasome inhibitor MG132 for 6 hours. Whole cell lysates were prepared by scraping cells into 95% ethanol at 4°C. Proteins extracted in the ethanol soluble fraction were separated on a 4–15% polyacrylamide gel and probed with anti-2,5P antibody. Following initial immunoblotting, the membrane was stripped and re-probed with anti-actin antibody to serve as a loading control. Since the CTD of human RNAPII contains 52 heptapeptide repeat units, UV-induced the cleavage of Rpb1 releasing a P-CTD fragment that was approximately twice the size (∼110 kDa) of the yeast P-CTD fragment which contains 25 heptapeptide repeats (∼50 kDa, see panel A). In repeated experiments, the P-CTD fragment was frequently observed in extracts from unirradiated MG132 treated cells (data not shown) however, detection of the P-CTD fragment was enhanced in extracts from similarly treated cells exposed to UV. Greatly reduced levels of P-CTD cleavage was also observed in the BRCA1 defective cell line MDA-MD-436 [57] following UV-irradiation and proteasome inhibition (data not shown). (D) UV-induced RNAPII P-CTD cleavage is BRCA1-dependent. Cells with a BRCA1-BRCT domain defect (HCC1937) were separately transfected with empty vector (PC) or vectors expressing truncated BRCA1 (BRCA1-ΔM) and full-length wild type BRCA1. At 48 hours post transfection cells were UV-irradiated, treated with MG132 and homogenized in ethanol as described above (panel C) and methods. Aliquots (10 ul) from the insoluble fraction resuspended in loading buffer were separated by SDS-PAGE and immunoblotted (IB) with the indicated antibodies as described above (panel C). The P-CTD cleavage fragment was only observed following ectopic expression of full-length wild type BRCA1. The faster mobility of the BRCA1 protein expressed from the BRCA1-ΔM construct is due to an in frame internal deletion of 518 amino acids which retains the RING and BRCT domains of BRCA1 [26]. BRCA1 expressed endogenously in the HCC1937 cells is detected as a lighter band with mobility similar to that of the ectopically overexpressed BRCA1. (E) A model for BRCA1 DNA damage surveillance during transcription. This model describes a BRCA1-dependent transcription-mediated checkpoint/surveillance system that is highly conserved among eukaryotes. The components of this pathway were identified as suppressors of BRCA1-induced lethality in yeast. Following DNA damage within actively transcribing genes, the elongating phosphorylated RNA pol II (form IIo) is stalled by DNA damage. In yeast, the Ctk1p kinase specifies the phosphorylation pattern of the CTD “recognized” by BRCA1. BRCA1 in the presence of specific co-factors including DSIF (Spt4p/Spt5p) and the yeast degradation factor Def1p is proposed to bind the P-CTD. This activates a protease activity that cleaves the CTD from the catalytic domain of Rpb1p (resulting in CTD-less form IIb). We propose this event is an upsteam signal that activates checkpoint arrest in G1. The checkpoint arrest signal remains “on” until prematurely terminated mRNA is exported from the nucleus utilizing Mlp1p, Mlp2p and numerous nuclear pore components to be degraded at the cytoplasmic processing body utilizing Ccr4p and Dhh1p. Following P-CTD cleavage, Rpb1p is targeted for ubiquitin-mediated degradation at the proteasome.

Since BRCA1 may subvert a conserved transcription associated damage pathway in yeast to exert its lethal effects, we examined if UV-irradiation, an efficient block to transcriptional elongation, could also induce P-CTD cleavage in yeast. Following a moderate dose of UV irradiation (120 J/m^2^), a transient 50 kDa P-CTD fragment was detected between 4 and 5 hours post irradiation (Fig. 6A).

Although the repeated RNAPII CTD sequence of yeast and human cells is highly conserved, the human CTD is approximately twice the length of the yeast CTD. Consistent with this larger size, following UV-irradiation of human breast epithelial cells and immunoprecipitation with the 2,5P antibody, we detected the appearance of a ∼110 kDa P-CTD cleavage fragment in MCF7 and T47D (BRCA1 +/+) cells (Fig. 6B). To confirm the identity of this CTD fragment, we used comparative immunoprecipitations with 2,5P and a second anti-CTD specific antibody 8WG16, which recognizes both unphosphorylated as well as partially phosphorylated RNAPII CTDs. These anti-CTD antibodies detected the 110 kDa CTD fragment in both of the BRCA1 (+/+) cell lines MCF7 and T47D (Fig. 6B). Interestingly, the P-CTD fragment can be observed in extracts from unirradiated cells (Fig. 6B) suggesting that P-CTD cleavage may result from RNAPII pausing and/or spontaneous damage during ongoing transcription. Since treatment with the proteasome inhibitor MG132 was required to detect increased levels of the UV-induced P-CTD fragment in MCF7 cells (Fig. 6B), this suggests that the P-CTD fragment is targeted by ubiquitination for rapid proteasome degradation following cleavage. Persistence of the P-CTD fragment was also enhanced in MG132 treated yeast cells expressing BRCA1 (Fig. 4F). These results suggest that damage-induced P-CTD cleavage is a conserved eukaryotic response.

### Damage-induced P-CTD cleavage is a BRCA1-dependent response

To determine if P-CTD cleavage was a BRCA1-dependent damage response, we UV-irradiated BRCA1 (+/+) and BRCA1 (−/−) breast epithelial cells with and without exposure to the proteasome inhibitor MG132 (Fig. 6C). RNAPII P-CTD cleavage was observed in UV-irradiated BRCA1 (+/+) cells (MCF7) but not in BRCA1 (−/−) breast cancer cells (HCC1937) following proteasome inhibition (Fig. 6C). Since treatment with MG132 was required to visualize the UV-induced P-CTD fragment in BRCA1 (+/+) cells, the P-CTD is likely eliminated rapidly via the proteasome pathway. To confirm that UV-induced P-CTD cleavage was a BRCA1-dependent process, we ectopically expressed either full-length (wild type) or an internal deletion (ΔM) BRCA1 construct in the BRCA1 mutant cell line HCC1937. Following UV-irradiation and proteasome inhibition with MG132, the P-CTD fragment was only detected in cells expressing the full-length wild type BRCA1 (Fig. 6D). Expression of the truncated BRCA1 construct (ΔM) containing an in frame internal deletion spanning amino acids 775 to 1203 [26] in HCC1937 cells did not support P-CTD cleavage following UV-irradiation and proteasome inhibition (Fig. 6D). Furthermore, using the N20 antibody that recognizes the N terminus of RPB1, we clearly visualized the reciprocal ∼180 kDa RPB1 IIb cleavage product (a fragment that cannot be detected with the anti-2,5P antibody) that arises following loss of the P-CTD after DNA damage in HCC1937 cells that express wild type BRCA1 (Figure S2). Taken together, these results demonstrate that UV-induced P-CTD cleavage is mechanistically linked to expression of the wild type BRCA1 protein in human breast epithelial cells.

## Discussion

In trying to define a fundamental role for BRCA1 in breast cancer, we took advantage of the evolutionary distance between humans and yeast to identify proteins and/or processes that have the ability to interact functionally with BRCA1 in yeast; these BRCA1-interacting partners were identified by finding yeast deletions that suppress BRCA1-induced lethality. For such “targets” to be biologically relevant, the yeast genes and human orthologs would most likely play roles in DNA damage responses and genome stability, and they would have an increased likelihood to physically interact with the BRCA1 protein. We present evidence that fulfills these criteria for proteins in the DSIF complex (SPT4/SPT5). Further, DSIF and other conserved suppressors of BRCA1-induced lethality may be grouped into a temporally linked mRNA signaling/decay pathway that responds to DNA damage encountered by RNAPII in actively transcribing genes at the elongation stage of transcription (Fig. 6E). Specifically, we identified BRCA1 suppressors that participate in mRNA transcription elongation as well as mRNA export and decay. Some of the suppressors (*CCR4* and *DHH1*) were first identified and characterized as IR resistance genes in a *RAD9*-dependent, checkpoint pathway required for G1/S transition following DNA damage [12]. This checkpoint role for *CCR4* and *DHH1* has been subsequently confirmed by other laboratories [28] [29] [30] [31] and a role for BRCA1 in G1/S checkpoint arrest following IR damage has been previously described [32]. Similarly, following overexpression of BRCA1 in human cells, a G1 arrest was observed [26].

The two yeast genes whose deletion most potently suppressed BRCA1-induced G1 arrest and lethality were those of the conserved transcription elongation factor Spt4p and the elongation phase-specific CTD kinase I (Ctk1-kinase), underlying the critical role that transcription elongation and RNAPII CTD phosphorylation play in BRCA1-mediated lethality. Furthermore, in agreement with previously described physical interactions observed between BRCA1 and human RNAPII [33] [34], BRCA1 was found to interact with the P-CTD of RNAPII in yeast resulting in P-CTD cleavage. It should be noted that the ethanol procedure used to prevent BRCA1 degradation extracts a relatively small fraction of the total cellular protein. Therefore, the amount of RNAPII that undergoes cleavage is unclear at this time.

Disease-associated mutations in BRCA1 that are expressed in this system neither reduce the viability of the yeast nor result in P-CTD cleavage suggesting that these events may be causally linked. Further support for the association of P-CTD cleavage and lethality comes from the rescue of both phenotypes in the *def1Δ* strain, previously shown to be defective for ubiquitin-mediated RNAPII degradation following DNA damage [13]. These results implicate BRCA1 in DNA damage-mediated degradation of elongating RNAPII via cleavage of the P-CTD. The highly conserved nature of the Rpb1p CTD suggested that similar interactions occur in human cells. Our results in human breast cancer cell lines fully support this interpretation. We have shown that P-CTD fragments exist in breast cancer cell lines, are stabilized by proteasome inhibition, and depend upon the presence of wild type BRCA1. Reconstitution of the damage-induced P-CTD accumulation following ectopic expression of wild type BRCA1 in the mutant HCC1937 cell line provides direct evidence for this process.

Results from ectopic expression in yeast of mutant BRCA1 proteins and from examination of human HCC1937 cells (expressing a carboxy terminal BRCA1 deletion) both point to the BRCT domain of BRCA1 as the mediator of P-CTD cleavage and associated lethality in yeast. Although the BRCA1-BRCT domain is necessary to promote P-CTD cleavage, it is not sufficient. For example, the BRCA1(ΔM) construct (that contains intact RING and BRCT domains) fails to reconstitute BRCA1-induced P-CTD cleavage following DNA damage. Furthermore, the BRCA1-ΔM construct can promote ubiquitination of intact RPB1 [26], suggesting that P-CTD cleavage and ubiquitination are separate processes. Moreover, the relative ease in transiently overexpressing the BRCA1-ΔM construct [26] compared to the wild type may be related to this defect in P-CTD cleavage.

A critical role for BRCA1 and its stochiometric binding partner BARD1 in ubiquitin-mediated degradation of phosphorylated RNAPII has been previously described *in vitro* [26] [27]. Deletion of *DEF1* inhibits BRCA1-mediated RNAPII CTD cleavage and degradation much in the same manner as ubiquitin targeted RNAPII degradation is inhibited following UV in *def1Δ* yeast [13]. This suggests that BRCA1 subverts a conserved RNAPII degradation response to DNA damage to induce lethality in yeast. Following DNA damage, Def1p may be essential to couple arrested RNAPII to the proteasome to facilitate its degradation [35].

BRCA1 associates preferentially with the hyperphosphorylated form of RNAPII [36] [27] [26] and appears to modulate RNAPII CTD phosphorylation levels [21]. Our finding that loss of the CTD specific kinase gene *CTK1* completely suppresses BRCA1-induced lethality indicates that BRCA1 interacts preferentially with the phosphorylated elongating form of the RNAPII specified by CTDK-I (*i.e*. phosphorylated on Ser2 and Ser5). The preferential interaction of BRCT domains with phosphoserine [37] suggests a potential physical interaction between BRCA1 and the P-CTD and predicts a critical role for the conserved human Ctk1p ortholog CRKRS (see *CTK1* in Table S1). Others have reported that, upon treatment with DNA damaging agents, the association of BRCA1 with RNAPII was disrupted suggesting a link between DNA damage signaling/repair and transcription [36]. The apparent damage-induced disruption of BRCA1 interaction with RNAPII may reflect degradation of RNAPII following DNA damage, dependent on specific co-factors such as DSIF as suggested in this study. The finding of enhanced phosphorylation and subsequent proteasome-mediated degradation of RNAPII that involves P-CTD cleavage following DNA damage in both yeast ([38] [39] (this study) and human cells [26] [27] (this study) further illustrates the important conserved role these ubiquitin-mediated degradation processes play in the BRCA1 damage response. Moreover, the finding that proteasome mediated degradation of BRCA1 occurs primarily in G1 [40] supports a spontaneous role for BRCA1 in degrading RNAPII in the absence of damage. Alternatively, spontaneous damage within transcriptionally active genes may account for the P-CTD cleavage observed in unirradiated breast epithelial cells (Fig. 6B).

Interaction of BRCA1 with the negative elongation factor NELF-B/COBRA1 [41] combined with the finding that COBRA1, which, as part of the NELF complex interacts with DSIF to negatively regulate RNAPII transcription [42] is fully supportive of our determination that the DSIF complex also interacts genetically and physically with BRCA1 and suggests a damage signaling role for both COBRA1 and DSIF in concert with BRCA1 and RNAPII. Our study supports (Fig. 6E) an emerging model that human cells employ the RNAPII holoenzyme complex during transcription elongation for DNA damage surveillance [43] [44] . In this respect, BRCA1 may act as a sensor of DNA damage to monitor stalling of the transcription apparatus at DNA lesions and, therefore, participates in a transcriptional checkpoint to signal the presence of lesions within actively transcribing genes [45]. As suggested by this study, a G1 checkpoint arrest signal might be maintained until subsequent transport of the prematurely terminated transcript for degradation at P-bodies and disassembly of the RNAPII-BRCA1-DSIF complex by ubiquitin-mediated degradation at the proteasome (Fig. 6E). Our finding that the human ortholog of Dhh1p (DDX6) interacts with BRCA1 in the cytoplasm at P-bodies following DNA damage also supports such a model (T. Westmoreland and J. Marks, unpublished). The determination that Dhh1p (and its human ortholog, DDX6) is an essential component of processing bodies that are sites of mRNA decay [46] [47] allows us to functionally link transcription elongation arrest by DNA damage to mRNA export and decay through BRCA1 and P-CTD cleavage (Fig. 6E). Thus BRCA1 may facilitate repair of DNA damage by mediating P-CTD cleavage and subsequent degradation of stalled transcription complexes to allow enhanced access of repair complexes to the lesion site. This model also accommodates reports that BRCA1 participates in transcription-coupled repair [48] [49] and is supported by the finding that overexpression of hTREX84 and dysregulation of mRNA export may be a hallmark of breast tumors [50]. Taken together our data support BRCA1 playing a critical role in a novel checkpoint pathway that senses DNA damage within actively transcribed DNA to initiate cleavage and degradation of stalled RNAPII elongation complexes. Furthermore, our results showing BRCA1-BRCT function is required for the rapid alkylation-induced degradation of BRCA1-SPT5 and RNAPII suggests that chemotherapeutic treatments utilizing alkylating agents may be clinically effective for treating breast cancers in which BRCA1-mediated degradation is defective. Moreover, the use of proteasome inhibitors in concert with alkylating agents may be equally effective for treating those breast cancers in which the damage-induced BRCA1-SPT5-RNAPII degradation pathway remains intact. Defects in this conserved BRCA1-dependent RNAPII cleavage and degradation pathway may be critical for the initiation of breast or ovarian cancer, predicting that genetic defects in other conserved components of this pathway may also contribute to these diseases.

## Materials and Methods

### Strain and plasmid constructs

Unless otherwise stated, WT and deletion strains are in the diploid BY4743 strain background. The strains, primers and technique utilized for deletion strain construction have been described (www-sequence.stanford.edu/group/yeast_deletion_project/deletions3.html). The YPH656 YAC containing strain has been previously described [51]. *SPT4* was deleted in the YPH656 strain by PCR amplifying the *spt4Δ::KanMX* loci from the *spt4Δ* strain in the BY4743 strain background obtained from the diploid deletion strain collection (Open Biosystems, Huntsville, AL) using forward (5′-GCG GTC CTC GTA GTC CAA TTT ACG TG-3′) and reverse (5′ GCC TTA GTT GAA TTA CTG GAC GGT AG-3′) primers that flank the *spt4Δ::KanMX* deletion sites. Further details of strain and plasmid constructions are in the supplementary Methods S1.

### Genome-wide screen for suppressors of BRCA1-induced lethality

A pool of 4746 nonessential diploid deletion strains each containing a unique 20 base pair genetic tag was obtained from Invitrogen (Carlsbad, CA). Yeast deletion strains were thawed from a frozen aliquot of the pooled strains (200 µl, at room temperature) and grown by inoculating the thawed cells into a 50 ml volume of YPD liquid medium maintained at 30°C with shaking until a cell count of 1-2×10^7^ cells/ml was obtained. Cells were made competent and transformed with 1-2 µg of the previously described [8] selectable (*URA3*) high copy *GAL*::BRCA1 YEp24 yeast BRCA1 expression plasmid (BRCA1 plasmid). Following transformation, cells were plated directly to synthetic complete (SC) galactose containing medium (2%) lacking uracil (GAL). Rapidly growing colonies were picked following 2–4 days incubation at 30°C. Individual colonies were streak purified on GAL plates. Individual colonies were tested for the ability to suppress BRCA1-induced lethality by growing WT and mutant strains containing the BRCA1 plasmid in liquid SC glucose containing medium (2%) lacking uracil (GLU) until stationary phase was reached in 96 well dishes. Stationary phase cultures were serially diluted (5 fold) in sterile water and 2 µl aliquots were replica plated to GLU and GAL solid medium. Isolates that showed enhanced survival on GAL plates when compared to WT were processed for identification [52]. To confirm that the identified deletions were true suppressors of BRCA1-induced lethality, we reestablished the BRCA1 plasmid into the individual deletion strains from the arrayed diploid deletion strain collection (Open Biosystems) and confirmed enhanced survival on GAL *vs* GLU.

### Suppression of BRCA1-induced G1 arrest and lethality

To examine suppression of the BRCA1-induced checkpoint delay at G1, WT, *spt4Δ, def1Δ* and *ctk1Δ* strains containing the BRCA1 plasmid were grown to logarithmic phase (1-2×10^7^ cells/ml) in liquid GLU medium. Single unbudded (G1) cells were micromanipulated into a 4×5 grid pattern of cells onto solid GLU and GAL plates using a Singer MSM 300 dissecting microscope (Singer Instrument Co., Somerset, UK) and photographed hourly.

### Western analysis of BRCA1 and RNAPII extracted from yeast or breast epithelial cells

To minimize degradation of BRCA1 and RNAPII during extraction from yeast, cell pellets from 50 ml of growth cultures were homogenized by bead beating in 0.7 ml of 95% ice cold ethanol on dry ice as previously described [53] with the following modifications. Cells were lysed by bead beating for 45 second pulses (6×) at maximum velocity in a Fastprep FP120 bead beater (Q-biogene, Irvine, CA). Lysates were allowed to cool at 4°C for 5 minutes between pulses. Insoluble material was pelleted and the resulting supernatant transferred to a new microfuge tube and evaporated to dryness under low heat in a rotary evaporator. Dried protein samples were resuspended in 2× SDS-PAGE loading buffer without tracking dye. Protein concentrations were determined using the NanoOrange detection kit (Invitrogen) before addition of tracking dye and separation by SDS-PAGE. Separated proteins were electroblotted to nitrocellulose and probed with antibodies against the hyperphosphorylated CTD of RNAPII largest subunit [“anti-2,5P”: is from a rabbit antiserum that was raised against a CTD fusion protein exhaustively phosphorylated by CTDK-I; IgGs were affinity purified (see [53]), first using the phosphorylated fusion protein and next using a synthetic CTD 3-repeat peptide phosphorylated on every Ser2 and Ser5 of the heptapeptide repeats (Ser2,5P; see [25])]. Anti-Act1p (sc-1615, Santa Cruz, Santa Cruz, CA) or anti-Pgk1p (anti-PGK 22C5, Invitrogen) were used as loading control probes. Following detection of RNAPII, blots were stripped and reprobed for BRCA1 (MS110, EMD Chemicals, San Diego, CA).

For mammalian cells, RNAPII was extracted by scraping cells directly into ice cold 95% ethanol (0.5 ml per 5×10^6^ cells). Cells were freeze-thawed three times and insoluble material was pelleted. The supernatant fluid was evaporated to dryness in a rotary evaporator and the resulting protein pellet resuspended in 2× loading buffer with β-mercaptoethanol. Alternatively, the pellet of ethanol insoluble material was resuspended in a large volume of 2× loading buffer (300 ul per 5×10^6^ cells). Aliquots (10–30 ul) were separated, electroblotted, and probed as described above.

### Exposure of yeast and mammalian cells to DNA damaging agents

WT and mutant yeast strains were grown to stationary phase at 30° (2 days) in 200 µl of YPD liquid medium in 96 well dishes. The cells were serially diluted in YPD (5 fold) and replica plated to YPD plates alone or YPD plates containing 0.02%–0.03% methyl methanesulfonate (MMS), 200mM hydroxyurea (HU) or 66% crude zymocin as previously described [12]. Cells plated to YPD alone were left unexposed or irradiated with 80 krads of γ-irradiation at a dose rate of 2.38 krads/min. Cells were continually exposed to HU, MMS or zymocin, by incubating the plates for 2–3 days at 30°. γ-ray survival curves were generated as previously described [12]. For UV-irradiation, WT diploid cells were grown to stationary phase as described above in 80 ml of YPD liquid medium. Unirradiated cells, (5 ml) were concentrated into a pellet by centrifugation and the supernatant fluid discarded. The cell pellet was flash frozen and stored as described above. A 40 ml volume of the remaining cell suspension was concentrated into a pellet by centrifugation, washed 1× in sterile H_2_O and resuspended in 15 ml of H_2_O. Cells were UV-irradiated in a sterile 100 mm Petri dish with constant agitation at a dose rate of ∼1 J/m^2^/sec to a total dose of 120 J/m^2^ using a germicidal lamp. Following irradiation cells were diluted to 40 ml in H_2_O and mixed with 40 ml of 2× liquid YPD. Cells were incubated at 30°C with vigorous agitation and 10 ml aliquots were concentrated by centrifugation, the supernatant was discarded and the resulting pellet was flash frozen at hourly intervals. Frozen cell pellets were extracted on dry ice in 0.4 ml 95% ethanol as described above. Extracted protein samples were resuspended in 50 ul of 2× loading buffer and 20 ul volumes separated on Tris-HCL gels as described above. RNAPII CTD cleavage was detected using anti-2,5P (P-CTD) antibody. Unirradiated and UV-irradiated cells were diluted in sterile water and plated to YPD plates to determine the relative survival of colony forming ability following UV-irradiation.

MCF7 and HCC1937 human breast cancer cell lines were grown to ∼80% confluence in RPMI 1640 medium containing 10% fetal bovine serum. Medium was removed from the cell monolayers and either left unirradiated or UV-irradiated (20 J/m^2^) using a UV Stratalinker 1800 (Stratagene, La Jolla, CA). Cells were refed with fresh medium with or without MG132 for 4–6 hours and whole cell lysates (WCL) prepared. For MMS treatment of human cells, MMS was placed in fresh complete RPMI medium and cells were incubated for 2 hours at 37°C.

### Southern blot analysis of BRCA1 plasmid stability in yeast

WT and *spt4Δ* strains containing the BRCA1 plasmid were grown to stationary phase and total genomic DNA extracted [54]. Equal aliquots of extracted DNA were separated on a 0.7% agarose gel, transferred to a nylon membrane by Southern blotting using a positive pressure apparatus (Stratagene) and immobilized by UV cross-linking as previously described [54]. The BRCA1 plasmid was labeled and visualized on the membrane following hybridization using the non-radioactive AlkPhos Direct labeling and detection kit (Amersham Biosciences, Piscataway, NJ).

### Proteasome inhibition in yeast and mammalian cells using MG132

The *erg6Δ* strain [55] in the BY4743 background containing the BRCA1 plasmid was induced for BRCA1 expression in GAL as described above. At 6 hours following GAL induction, cells were treated with 50 µM MG132 or vehicle (DMSO). Undamaged MCF7 or HCC1937 cells or those exposed to UV (20 J/m^2^) were concomitantly treated with MG132 (dissolved in DMSO) resuspended in complete RPMI medium to a final concentration of 50 µM.

### Co-immunoprecipitation

To prepare yeast WCL, cells were concentrated by centrifugation and residual growth medium discarded. Cell pellets were resuspended in 0.5 ml of ice cold NP40 buffer (0.05–0.1% NP40, 50 mM Tris pH 8.0, 5 mM EDTA, 150 mM NaCl) plus protease inhibitors and lysed by bead beating for 45 second pulses at maximum velocity in a Fastprep FP120 bead beater (Q-biogene, Irvine, CA). Lysates were allowed to cool at 4°C for 5 minutes between pulses. WCL were precleared by centrifugation followed by incubation of the supernatant for 24 hrs at 4°C normal mouse IgG (Vector Laboratories, Burlingame, CA) plus a 1∶1 mixture of Protein A and G beads (Roche Diagnostics, Nutley, NJ). For co-IP interactions, precleared lysate was reacted overnight at 4°C with normal mouse IgG, anti-V5 (Invitrogen) or anti–BRCA1 antibody (MS110) plus Protein A and/or G agarose. IP reactions were washed 2-3× in NP40 buffer with protease inhibitors. Following the last wash, SDS-PAGE loading buffer was added to the beads and heated at 95°C for 5 minutes prior to SDS-PAGE.

For breast cancer cell lines, culture monolayers were gently washed 3× in ice cold PBS and lysed with gentle shaking at 4°C in 0.5 ml of NP40 buffer (0.08% NP40) plus protease inhibitors. WCL were precleared with normal mouse IgG and subjected to co-IP analysis as described above using anti-RNAPII CTD antibody, anti-BRCA1 antibody (MS110), or anti-SPT5 antibody (anti-DSIF 61107, BD Biosciences, San Jose, CA). For detection on Western blots; N20 (sc-899, Santa Cruz) or anti-2,5P were used as probes.

### Immunofluorescent detection of BRCA1 in yeast

IF detection of BRCA1 in yeast was adapted from previously described methods [56]. Briefly, after BRCA1 expression was induced by shaking for six hours at 30°C in GAL, cells were fixed in formaldehyde for 1 hr at RT, recovered by centrifugation, resuspended in 50 mM phosphate-buffered formaldehyde plus sorbitol (1.2M) and fixed a further two hours at RT. After Zymolyase treatment (1 mg/ml Zymolyase 100T ICN) for 30 min at 37°C, cells were washed and stored at 4°C for antibody staining. For BRCA1 detection, cells were placed onto polylysine-coated slides. The cells were fixed again at −20°C in methanol and acetone and then blocked (5% normal goat serum, 1% glycerol, 0.1% BSA, 0.1% fish skin gelatin in sterile PBS) for 30 minutes. The primary antibody (MS1101 µg/ml) was reacted for 2 hrs at RT, followed by incubation for 1 hr with a goat anti-mouse IgG conjugated with Alexflour 488 (Jackson Immunoresearch Laboratories, West Grove, PA). Hoechst 33342 diluted 1∶10,000 in PBS was added 5 minutes before coverslipping.

## Supporting Information

Table S1 Identification of yeast diploid deletion strains that suppress lethality following heterologous expression of the tumor suppressor BRCA1.
(0.15 MB DOC)

Figure S1 Characterization of the BRCA1-induced P-CTD fragment. The 50 kDa BRCA1-induced fragment detected by anti 2,5P is specific for the RNAPII CTD. The yeast strain CTD-His10a expresses an Rpb1p allele with an in frame C-terminal His10 tag fused to the Rpb1p CTD (left panels). The 12AR yeast strain expresses Rpb1p with a truncated CTD containing 12 heptapeptide repeats. These strains were induced for BRCA1 expression and extracted in ethanol as described above. For the CTD-His10a strain, Western blots were probed with anti-2,5P P-CTD antibody (upper panels), stripped and reprobed with anti-His antibody (Amersham, lower panel). The anti-His antibody detects the 50 kDa fragment previously detected by the anti-2,5P CTD antibody. The yeast strain expressing a truncated RNAPII CTD (12AR) exhibited a smaller P-CTD fragment when compared to the BRCA1-induced fragment from the WT strain containing the full-length Rpb1p CTD using the anti-2,5P antibody as a probe. Lower panel is a longer exposure of the upper panel.
(7.72 MB TIF)

Figure S2 DNA damage induces BRCA1-dependent cleavage of RPB1 in breast epithelial cells. Human breast epithelial cells that are mutant for BRCA1 (HCC1937) were transfected with PC control, BRCA1-delta M and wild-type BRCA1 as described in methods and Fig. 6D. Following transfection, cells were UV-irradiated (20 J/m^2^) and treated with the proteasome inhibitor MG132 for 6 hours. Sample aliquotes (30 ul) identical to that described in Fig. 6D were separated by SDS-PAGE and transferred to nitrocellulose. The Western blot was probed with the N20 antibody specific for the N terminus of RPB1. Following initial immunoblotting (see Fig. 6D), stripped membranes were reprobed with anti-actin antibody to serve as a loading control. The majority of full-length RPB1was converted from the hypophosphorylated from of RPB1 (IIa) to the hyperphosphorylated form of Rpb1 (IIo). UV damage induced a BRCA1-dependent cleavage of RPB1 into the IIb form (∼180 kDa) and the P-CTD fragment (see Fig. 6D) that is not detected by the N20 antibody.
(3.75 MB TIF)

Methods S1 (0.05 MB DOC)
