# Supplementary material for: Yeast Screens Identify the RNA Polymerase II CTD and SPT5 as Relevant Targets of BRCA1 Interaction
Source: PLoS One. 2008 Jan 16;3(1):e1448. doi: 10.1371/journal.pone.0001448 (PMC2174531; doi:10.1371/journal.pone.0001448)
Supplement: Table S1 — Identification of yeast diploid deletion strains that suppress lethality following heterologous expression of the tumor suppressor BRCA1. (0.15 MB DOC) [file pone.0001448.s001.doc]

Table S1. Identification of yeast diploid deletion strains that suppress lethality following heterologous expression of the tumor suppressor BRCA1.

| **Deleted yeast gene/**  **ORF** | **Screen^1^** | **Function in Yeast Cells** | **BRCA1**  **Interaction Network^2^** | **Human Ortholog**  (P value)**^3^** | **Function in Human Cells** | GAL-URA^4^ | GALGLU^5^ | IR^6^ | **Zymocin sensitivity^7^** |
| --- | --- | --- | --- | --- | --- | --- | --- | --- | --- |
| *Suppressors of BRCA1-induced lethality identified from a random pool of diploid deletion mutants* | | | | | | | | | |
| *ate1* | Pool | Arginyl-tRNA-protein transferase,: required for degradation via the N-end rule pathway | - | ATE1 (-23) | arginyltransferase 1 | ++ | **0.35** | S | - |
| *bbc1*(x2) | Pool | Actin cytoskeletin organization | Spt5p *bem4*  *rvs161*  *rvs167* | MUC7(-22) | mucin 7, salivary, secreted | +++ | **0.69** | S | ++++ |
| ***bem4*** (x2) | Pool | Bud emergence | *bbc1* | - | - | ND | **0.25** | ND | ND |
| ***dan1*** | Pool | Anaerobic induced cell wall mannoprotein | - | LOC394263(-13) | unknown | + | **0.28** | S | +++ |
| ***gyp5***  (x3) | Pool | GTPase-activating protein for Rab | Rvs161p Rvs167p | RAB6A(-51) | RAB GTPase activating protein 1 | ++ | **0.34** | S | +++ |
| ***hda3*** | Pool | Histone deacetylase | - | GCC2(-08) | GRIP and coiled-coil domain containing protein 2, localized to the trans-Golgi network | ++ | **0.42** | S | ++++ |
| *mlp2* | Pool | Myosin like protein nuclear import/export | Mlp1p *mlp1*  *nup120*  *nup133* | TPR(-64) | translocated promoter region (to MET oncogene) | ++ | **0.46** | - | ++ |
| *npr1* | Pool | Protein kinase | - | CHEK1(-22) | CHK1 checkpoint homolog (*S. pombe*) | ++ | **0.57** | S | ++++ |
| *nup2* (x2) | Pool | Nuclear pore protein: guidance and recycling of nuclear transport receptors | *nup120* *nup170* | RANBP2 (-18) | GTP-binding protein at the nuclear membrane | +++ | **0.54** | S | ++ |
| ***pom34*** (x2) | Pool | Nuclear pore protein; membrane associated | *nup133*  *nup120* | - | - | +++ | **0.50** | - | ++ |
| *spt4* | Pool | Protein that forms a complex with Spt5p and mediates both activation and inhibition of transcription elongation | Rpb1p*Spt5p* Rpb4p  *rpo21*  *ctk1* | SUPT4H1 (-18) | Suppressor of Ty 4 homolog 1, member of DSIF transcription complex | ++++ | **1.21** | S | ++++ |
| *sub1* | Pool | Transcriptional co-activator facilitates elongation by influencing enzymes that modify RNAPII | Spt5p Sua7p*  *sua7* | SUB1(-12) | SUB1 homolog, activated RNA pol II transcription cofactor 4 | ++ | **0.34** | S | ++++ |
| *tpm1* | Pool | Tropomyosin, required for actin cable stability | - | TPM2 (-15) | Tropomyosin 2 (beta) | ++ | **0.54** | S | ++++ |
| *yaf9* | Pool, IR | Subunit of both the NuA4 histone H4 acetyltransferase complex and the SWR1 complex, chromatin modifying | *ccr4* | YEATS4(-32) | YEATS domain containing 4, homology to MLLT1/ 3 transcription factors | ++ | **0.54** | S | ++++ |
| ***yap3*** | Pool | Basic leucine zipper (bZIP) transcription factor | Nup116p* | XBP1 (-05) | X-box binding protein 1; over-expression in human breast cancer | ++ | **0.35** | SS | ++++ |
| *yal042c-a* | Pool | Dubious ORF: deletion overlaps 5’ end of ***ERV46*** | - | - ERGIC3 (-67) | -  Serologically defined breast cancer antigen 84 isoform a | + | **0.25** | - | ++ |
| ***ygr053c*** | Pool | Uncharacterized ORF: interacts with transcription factor Sua7p | Sua7p* | - | - | + | **0.32** | - | ++ |
| ***ygr064w*** (x2) | Pool | dubious ORF: deletion overlaps 5’ end of ***SPT4*** | - Rpb1p*Spt5p* Rpb4p  *rpo21*  *ctk1* | - | - | +++ | **1.53** | S | +++ |
| ***yjr014w*** (x2) | Pool | ***TMA22***: translation machinery associated; RNA binding | Spt5p* | DENR(-22) | translation initiation factor; increased expression in breast cancer cells that overexpress HER-2/neu proto-oncogene | + | **0.26** | - | ++ |
| ***ymr172c-a*** | Pool | dubious ORF: deletion overlaps the 3’ end of ***HOT1*** | - | - DSPP (-09) | -  Dentin sialophosphoprotein: Expression in human prostate cancer correlates with tumor aggressiveness | + | **0.26** | - | ++ |
| *Suppressors of BRCA1-induced lethality identified by screening diploid deletion strains sensitive to ionizing radiation* | | | | | | | | | |
| *asm4* | Network prediction  IR | Nuclear pore protein: part of a subcomplex also containing Nup53p, Nup170p, and Pse1p | Nup53p, Nup170p | DSPP(-06) | Expression of dentin sialophosphoprotein in human prostate cancer correlates with tumor aggressiveness | ND | **0.37** | S | ND |
| ***bur2*** | IR | Cyclin for the Sgv1p protein kinase; involved in phosphorylation of the carboxy terminal domain (CTD) of the major subunit of RNAP II | *spt4* | - | - | ++ | **0.31** | S | ND |
| ***ccr4*^8^** | IR | CCR4-NOT transcription complex member, major cytoplasmic deadenylase, localized to cytoplasmic P-bodies | Dhh1p*dhh1**spt5* *yaf9*  *rvs161*  *ctk1* | CNOT6KIAA1194 (-83) | CCR4-NOT transcription complex, subunit 6 | ++++ | **0.59** | SS | ++++ |
| ***def1*** | IR | RNAP II degradation factor required for ubiquination and subsequent degradation of Rpb1p following DNA damage | Rpb1p**rpo21**rad16* | IVL (-16) | involucrin | +++ | **0.81** | SSS | ++++ |
| ***dhh1*^8^** | IR | CCR4-NOT transcription complex member, RNA DEAD box helicase, stimulates mRNA decapping, and decay, localized to cytoplasmic P bodies | Ccr4p *ccr4* | DDX6 (-162) | RNA DEAD box helicase, overexpressed in colorectal cancer, localized to cytoplasmic P bodies | +++ | **0.60** | SSS | ++++ |
| ***nup120*** | IR | Nuclear pore protein: involved in nuclear poly(A)+ RNA export | Nup188p  *nup133*  *nup170*  *nup2*  *pom34*  *mlp1* | - | - | +++ | **0.63** | SSS | ND |
| ***nup133*** | IR | Nuclear pore protein: involved in nuclear poly(A)+ RNA export | Nup188p  *nup120*  *mlp2*  *nup188*  *nup170*  *nup2*  *pom34* | - | - | ND | **0.20** | SSS | ND |
| ***nup170*** | IR | Nuclear pore protein: required for chromosome transmission fidelity | Nup53p Asm4p  *nup2*  *nup133*  *nup120* | NUP155 (-30) | Nucleoporin 155 kDa | +++ | **0.70** | S | ND |
| ***nup188*** | IR | Nuclear pore protein | Nup133p Nup120p  *nup133*  *nup120* | NUP188 (-04) | Nucleoporin 188 kDa | +++ | **0.38** | S | ND |
| *rad50*^9^ | IR | Subunit of MRX complex, with Mre11p and Xrs2p, involved in processing double-strand DNA breaks and non homologous end joining repair of DSBs | Xrs2p *nup133*  *nup120*  *ccr4*  *ctk1* | RAD50 (-157) | RAD50 homolog (*S. cerevisiae*) | ND | **0.36** | SSS | +++ |
| *rad51*^9^ | IR | Strand exchange protein, forms a helical filament with DNA that searches for homology; involved in the recombinational repair of double-strand breaks in DNA | Rad52p *ccr4*  *nup120*  *nup133*  *ctk1* | RAD51 (-130) | RAD51 homolog (RecA homolog, *E. coli*) (*S. cerevisiae*) | ++ | **0.25** | SSS | ++ |
| *rad52*^9^ | IR | Protein that stimulates strand exchange by facilitating Rad51p binding to single-stranded DNA; anneals complementary single-stranded DNA; involved in the repair of double-strand breaks | Rad51p *ccr4*  *nup120*  *nup133*  *ctk1* | RAD52(-40) | RAD52 homolog (*S. cerevisiae*) | + | **0.27** | SSS | +++ |
| *rad55*^9^ | IR | Involved in the recombinational repair of double-strand breaks in DNA during vegetative growth and meiosis | Rad51p *rad51*  *rad52*  *ccr4*  *nup120*  *nup133*  *ctk1* | RAD51L3 (-05) | RAD51-like 3 (*S. cerevisiae*) | ND | **0.22** | SSS | +++ |
| *xrs2*^9^ | IR | Protein required for repair of double strand breaks, meiotic recombination, telomere maintenance, and checkpoint signaling | Rad50p *rad50*  *nup120*  *nup133*  *ccr4*  *ctk1* | DSPP (-05) | Dentin sialophosphoprotein: Expression in human prostate cancer correlates with tumor aggressiveness | ND | **0.32** | SSS | +++ |
| ***yml009c-a*** | IR | Dubious ORF: deletion overlaps the 3’ end of ***SPT5*** | -  Rpb1p*  *rpo21*  *ctk1* | - SUPT5H (-83) | -  Suppressor of Ty 5 human ortholog member of DSIF transcription complex | ++ | **0.44** | SS | ND |
| ***yml009w-b*** | IR | Dubious ORF: deletion overlaps the 3’ end of ***SPT5*** | -  Rpo21p*  *rpo21*  *ctk1* | - SUPT5H (-83) | -  Suppressor of Ty 5 human ortholog member of DSIF transcription complex | + | **0.35** | S | ND |
| ***Suppressors of BRCA1-induced lethality identified by screening mutants predicted to be components of the mRNA export and decay pathway*** | | | | | | | | | |
| ***ctk1*** | Network prediction | Catalytic (alpha) subunit of C-terminal domain kinase that phosphorylates the CTD domain Rpb1p to promote transcription elongation. | Rpb1p* *ccr4*  spt4  *spt5* | CRKRS (-73) | Cdc2-related protein kinase 7; arginine/serenine (RS) rich | ND | **1.23** | ND | ND |
| *met18*^10^ | Network prediction | DNA excision repair and TFIIH regulator, required for RNAP II transcription | Rvs167p | MMS19L (-27) | MMS19-like (MET18 homolog, S. cerevisiae) | ND | **0.25** | ND | ND |
| *mlp1* | Network prediction | Myosin-like protein nuclear import/export | Mlp2p *mlp2*  *nup120* | TPR (-113) | translocated promoter region (to MET oncogene) | ND | **0.47** | ND | ND |
| ***nup53*** | Network prediction | Nuclear pore protein; interacts with karyopherin Kap121p or with Nup170p | Asm4p  Nup170p | **NUP35**  (-05) | Nucleoporin 35kDa | ND | **0.38** | ND | ND |
| *rad16*^10^ | Network  prediction | subunit of Nucleotide Excision Repair Factor 4 (NEF4); member of the SWI/SNF family | *def1* | SMARCA3 (-75) | SWI/SNF related, matrix associated, actin dependent regulator of chromatin, subfamily a, member 3 | ND | **0.29** | ND | ND |
| *rpb4*^10^ | Network  prediction | RNAP II subunit B32; forms two subunit dissociable complex with Rpb7p; involved in export of mRNA to cytoplasm under stress conditions | Rpb1p*Spt5p* Spt4p | POLR2D (-12) | Polymerase (RNA) II (DNA directed) polypeptide D | ND | **0.29** | ND | ND |
| *sli15*^11^ | Network prediction | Subunit of the Ipl1p-Sli15p-Bir1p complex that regulates kinetochore-microtubule attachments, also regulates the activity and localization of the Ipl1p aurora kinase | - | INCENP | inner centromere protein antigen | ND | **0.48** | ND | ND |
| ***srb10*** | Network prediction | Cyclin-dependent protein kinase, component of RNAP II holoenzyme; involved in phosphorylation of the RNAP II C-terminal domain | Ccr4p  Rpb1p*  *ctk1*  *spt4*  *spt5**  *rpb1** | **CDK8**  (-67) | cyclin-dependent kinase 8 | ND | **0.23** | ND | ND |
| ***ybp2*^11^** | Network prediction | Protein with a role in resistance to oxidative stress; similari to Ybp1p, which is involved in regulation of the transcription factor Yap1p via oxidation of specific cysteine residues | Rvs161p  Rvs167p | - | - | ND | **0.61** | ND | ND |

^1^ Deletions were identified by screening the tagged deletion strain pool (Pool) following transformation of the high copy selectable *GAL::*BRCA1 expression plasmid.. Strains were identified by sequencing PCR amplified identification tags adjacent to the G418 marker used to construct the deletion strains. Some deletion strains were independently identified more than once. For example, *gyp5Δ* was identified three times (x3) in separate transformation screens. Some strains were identified by screening the collection of ionizing radiation sensitive diploid deletion mutants described in Bennett et al. 2001 and Westmoreland et al.,2004. Preliminary characterization of the *ccr4Δ* and *dhh1Δ* suppressors of BRCA1 induced lethality have been previously described. The remaining deletion strains (Network prediction) were identified due to previously published network interactions with mutants that were experimentally confirmed to suppress BRCA1-induced lethality.

^2^Protein-protein (non-italicized) and genetic (italicized) interactions were identified from the SGD website. Proteins with an asterix (*) denote essential gene products.

^3^ P value as calculated by Blast analysis of yeast protein product against the human genome protein reference sequences. Gene assignments for human orthologs of yeast gene suppressors of BRCA1-induced lethality were determined using the currently accepted Human Genome Organization (HUGO) designation.

^4^ Enhanced suppression of growth inhibition (as compared to WT) after 3 days growth at 30^0^C on SC GAL-URA plates (BRCA1 expressed) using a dilution replica plating technique: + = 5-fold; ++ = 25-fold; +++ = 125 fold; ++++ = >500-fold.

^5^Relative plating efficiency of colony forming ability on SC GLU-URA (BRCA1 repressed) *vs* SC GAL-URA (BRCA1 expressed). The mean relative survival for WT strains containing the *GAL::*BRCA1 plasmid on GAL-URA *vs* GLU-URA was 0.098 + 0.08 (+ 1 standard error; n = 33). The mean survivals of listed suppressors of BRCA1 induced lethality were determined for 3 or more replica experiments and each mean listed was significantly greater than that described for WT (*i.e.* significantly greater than 0.19).

^6^ Deletion strains that showed at least a 5-fold (S) enhanced sensitivity to the killing effects of IR when compared to WT have been shown. Some strains may demonstrate slight (2-3 fold) sensitivity to IR (NT = not tested).

^7^Enhancement growth inhibition following exposure to zymocin (as compared to WT) after 3-4 days growth at 30^0^C on YPD + zymocin (66%) plates. A dilution replica plating technique was used as described in the legend to Fig. 3: + = 5-fold inhibition; ++ = 25-fold; +++ = 125 fold; ++++ = >500-fold.

^8^ Ccr4p and Dhh1p are two components of the CCR4-NOT transcription complex. We examined whether deletions of other members of this complex that are also IR sensitive could suppress BRCA1-induced lethality. Strains individually deleted for other CCR4-NOT complex members (*NOT4, NOT5, POP2* and *DBF2*) all failed to suppress BRCA1-induced lethality. Deletion of either *NOT4* or *NOT5* significantly enhanced BRCA1-induced lethality (>20 fold as compared to WT, data not shown). Deletion of either *POP2* or *LSM1*, both of which have been identified as components of cytoplasmic P bodies, failed to suppress BRCA1-induced lethality indicating that Dhh1p and Ccr4p have specialized key roles related to their functions associated with mRNA decay that also mediates resistance to IR damage.

^9^ In human cells, BRCA1 has also been shown to directly interact with components of the recombinational repair apparatus (RAD51) as well as components of the non-homologous end-joining pathway. We therefore examined strains individually deleted for members of the RAD52 epistasis group to determine if lethality was suppressed following GAL-induced expression of BRCA1. Little (*i.e.* *rad50Δ, rad51Δ, rad52Δ,* *rad55Δ * and *xrs2Δ*)* *or no suppression was observe

d for *rad54Δ, rad55Δ, rad57Δ* or *rdh54Δ* strains (data not shown) indicating recombinational repair is not a major suppressor pathway of BRCA1-induced lethality.

^10^ Since *DEF1* and *SPT4* have been linked to transcription coupled repair (TCR), we examined a number of deletion strains that have been implicated in TCR to determine if any were required for BRCA1-induced lethality. Little (*rad16Δ, met18Δ*, *rpb4Δ*) or no (*rad7Δ*, *rad23Δ, rad26Δ, rad28Δ*, *rpb9Δ*, *elc1Δ*; data not shown) suppression of BRCA1-induced lethality was observed indicating that TCR repair is not a major suppressor pathway of BRCA1-induced lethality.

^11^ In a separate screen, deletions of either *SLI15* or *YBP2* were found to suppress the lethal effects of heterologous expression of the breast cancer associated AuroraA kinase (C. Blanchette, C. Bennett and J. Marks, unpublished). Individual deletions of these two mutants also partially suppressed BRCA1-induced in yeast. Interestingly, similar to *not4Δ* and *not5Δ*, individual deletions of the IR resistance genes *RVS161* or *RVS167*, whose protein products physically interact with Ybp2p, were found to significantly enhance BRCA1-induced lethality in yeast (*i.e.* a 10 and 30 fold decrease in relative plating efficiency on GAL *versus* GLU for *rvs161Δ* and *rvs167Δ* when compared to WT strains containing *GAL::*BRCA1; data not shown). Sli15p contains a C-terminal INCENP_ARK (Aurora kinase) binding domain and is the ortholog of human INCENP although BLAST analysis as described above (3) failed to identify significant homology.
