# Supplementary figures and images for: Yeast Screens Identify the RNA Polymerase II CTD and SPT5 as Relevant Targets of BRCA1 Interaction
Source: PLoS One. 2008 Jan 16;3(1):e1448. doi: 10.1371/journal.pone.0001448 (PMC2174531; doi:10.1371/journal.pone.0001448)

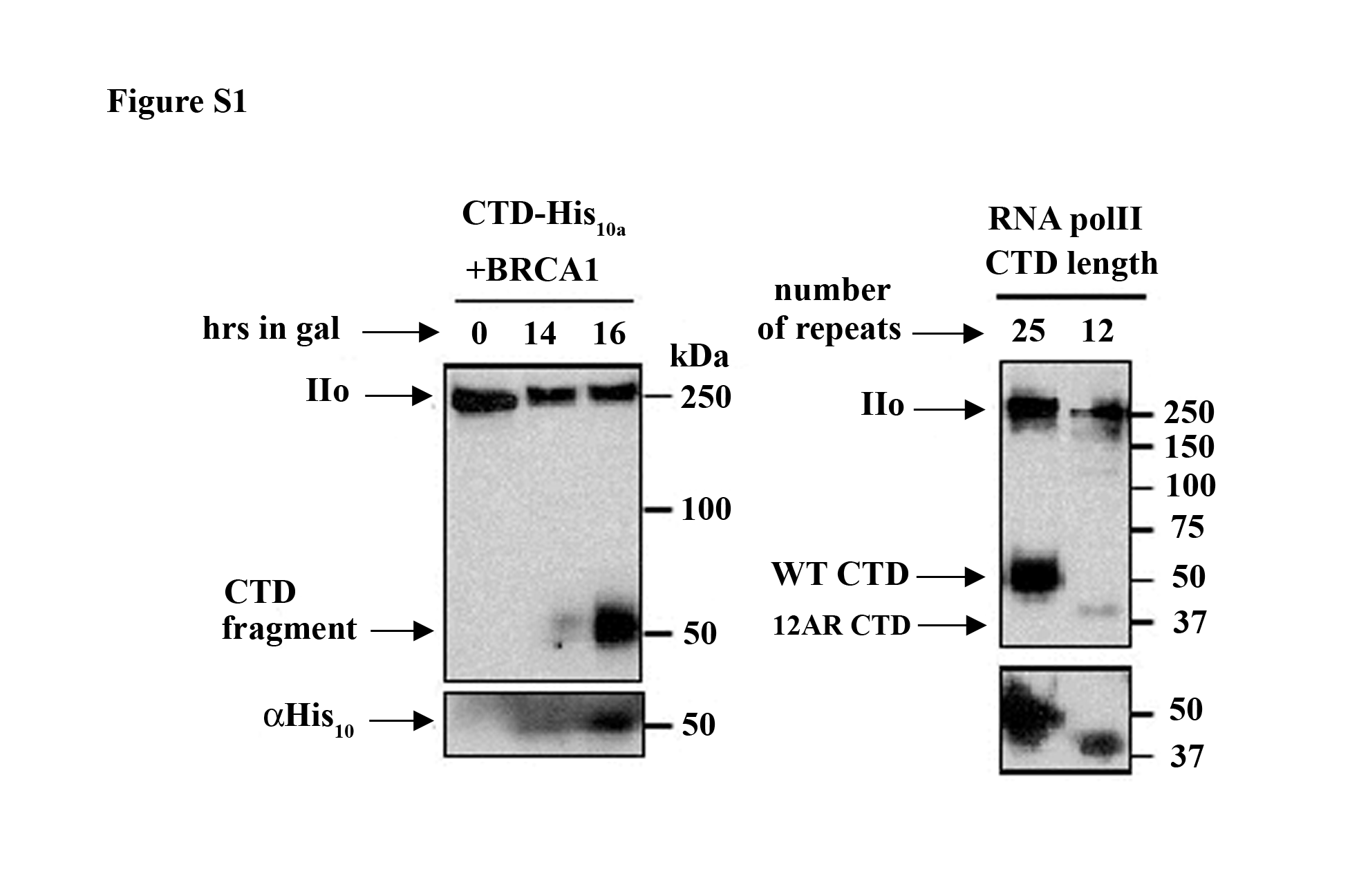

Supplement: Figure S1 — Characterization of the BRCA1-induced P-CTD fragment. The 50 kDa BRCA1-induced fragment detected by anti 2,5P is specific for the RNAPII CTD. The yeast strain CTD-His10a expresses an Rpb1p allele with an in frame C-terminal His10 tag fused to the Rpb1p CTD (left panels). The 12AR yeast strain expresses Rpb1p with a truncated CTD containing 12 heptapeptide repeats. These strains were induced for BRCA1 expression and extracted in ethanol as described above. For the CTD-His10a strain, Western blots were probed with anti-2,5P P-CTD antibody (upper panels), stripped and reprobed with anti-His antibody (Amersham, lower panel). The anti-His antibody detects the 50 kDa fragment previously detected by the anti-2,5P CTD antibody. The yeast strain expressing a truncated RNAPII CTD (12AR) exhibited a smaller P-CTD fragment when compared to the BRCA1-induced fragment from the WT strain containing the full-length Rpb1p CTD using the anti-2,5P antibody as a probe. Lower panel is a longer exposure of the upper panel. (7.72 MB TIF) [file pone.0001448.s002.tif]

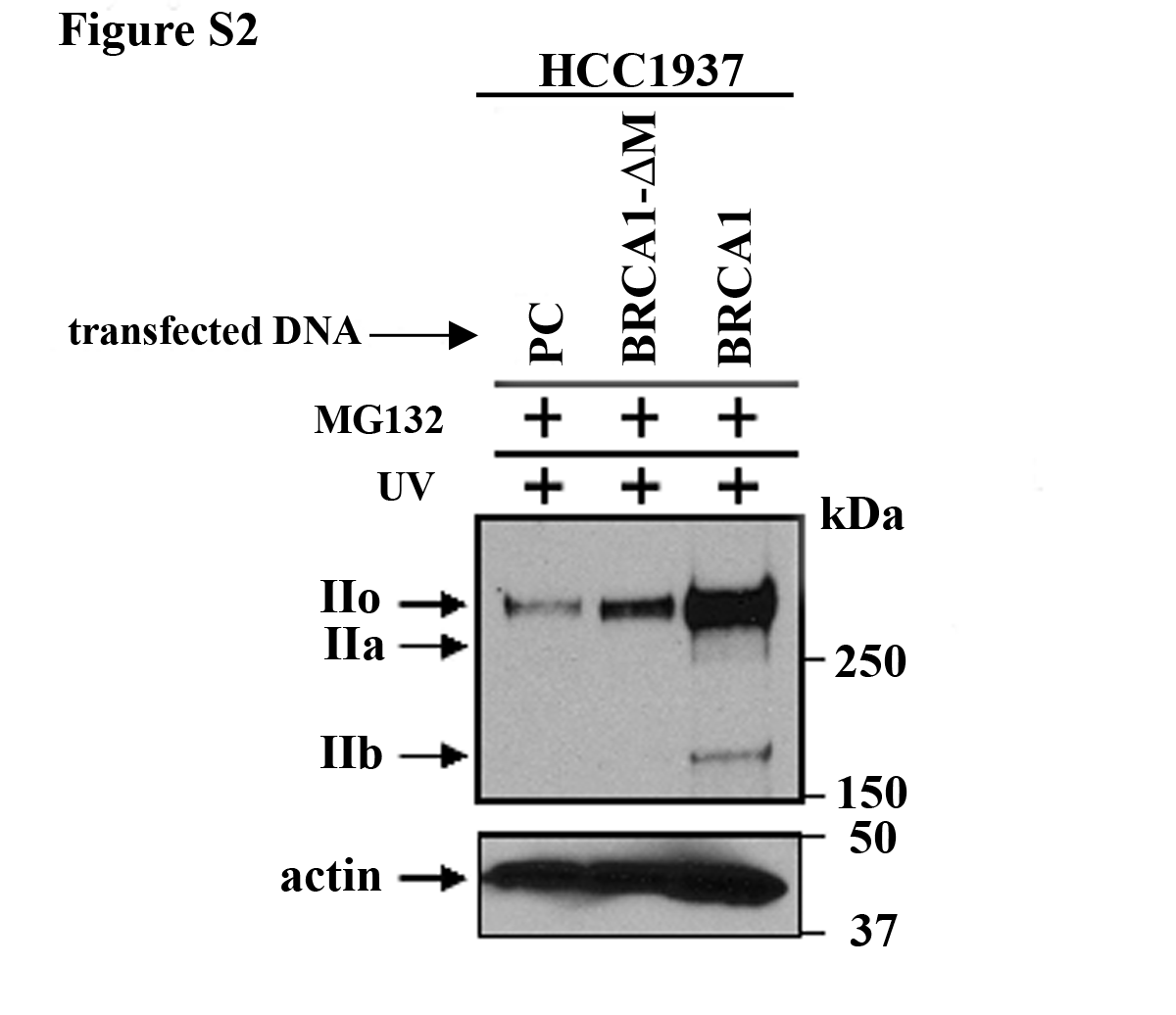

Supplement: Figure S2 — DNA damage induces BRCA1-dependent cleavage of RPB1 in breast epithelial cells. Human breast epithelial cells that are mutant for BRCA1 (HCC1937) were transfected with PC control, BRCA1-delta M and wild-type BRCA1 as described in methods and Fig. 6D. Following transfection, cells were UV-irradiated (20 J/m2) and treated with the proteasome inhibitor MG132 for 6 hours. Sample aliquotes (30 ul) identical to that described in Fig. 6D were separated by SDS-PAGE and transferred to nitrocellulose. The Western blot was probed with the N20 antibody specific for the N terminus of RPB1. Following initial immunoblotting (see Fig. 6D), stripped membranes were reprobed with anti-actin antibody to serve as a loading control. The majority of full-length RPB1was converted from the hypophosphorylated from of RPB1 (IIa) to the hyperphosphorylated form of Rpb1 (IIo). UV damage induced a BRCA1-dependent cleavage of RPB1 into the IIb form (∼180 kDa) and the P-CTD fragment (see Fig. 6D) that is not detected by the N20 antibody. (3.75 MB TIF) [file pone.0001448.s003.tif]
