# Supplementary material for: Yeast Screens Identify the RNA Polymerase II CTD and SPT5 as Relevant Targets of BRCA1 Interaction
Source: PLoS One. 2008 Jan 16;3(1):e1448. doi: 10.1371/journal.pone.0001448 (PMC2174531; doi:10.1371/journal.pone.0001448)
Supplement: Methods S1 — (0.05 MB DOC) [file pone.0001448.s004.doc]

# Methods S1

**Construction of the *GAL::SPT4*-FLAG-*LEU2* expression plasmid.** All plasmids were created using the Gateway^TM^ recombination-based cloning system (Invitrogen). PCR amplifications for cloning *SPT4* were carried out using Platinum Taq High Fidelity (Invitrogen). A yeast *SPT4* clone (from F. Winston) was amplified using primers OCB146/OCB148 and cloned into pDONR-221 to create the pENTR-ScSPT4 entry clone using BP clonase as per manufacturers instructions (Invitrogen). The pGAL1-C-FLAG destination vector (from M. Tyers) is a low-copy centromeric Gateway compatible selectable (*LEU2*) plasmid that incorporates a C-terminal FLAG tag epitope. The scSPT4 sequence from pDONR-221 was transferred to the pGAL1-C-FLAG destination vector using LR clonase as per manufacturers instructions (Invitrogen). All entry clones were sequenced over the entire length of the insert to ensure wild type sequence and all clones lack a stop codon to permit the creation of C-terminal fusion proteins.

OCB146: 5’-GGG GAC AAG TTT GTA CAA AAA AGC AGG CTT CGA AGG AGA TGT GTA AGA GAT GTC TAG TGA-3’

OCB148: 5’-GGG GAC CAC TTT GTA CAA GAA AGC TGG GTC CTC AAC TTG ACT GCC

ATC-3’

The *GAL::SPT4*-FLAG-*LEU2* expression plasmid along with the *GAL::*BRCA1-*URA3* plasmid was established by transformation within the diploid *spt4Δ* strain. Co-expression of Spt4p and BRCA1 reestablished BRCA1-induced lethality (see Fig. 1F in manuscript).

Construction of the *GAL::SPT4*-V5-*URA3* high copy expression plasmid. The construction strategy described above was used to place the scSPT4 sequence from pDONR221 into the pDEST52 destination vector using Gateway cloning and LR clonase as per manufacturers instructions (Invitrogen). The *GAL::SPT4*-V5-*URA3* plasmid was established along with the *GAL::*BRCA1-*HIS3* plasmid by transformation within the wild type (WT) diploid BY4743 strain. Co-expression of Spt4p-V5 and BRCA1 resulted in physical interaction as determined by co-IP analysis (see Fig. 5A in manuscript).

Construction of the *GAL::*BRCA1-*HIS3* expression plasmid. The *URA3* selectable marker of the *GAL::*BRCA1 Yep24 derived plasmid was replaced with the *HIS3* selectable marker using an *in vivo* recombinational cloning approach. The *HIS3* gene was PCR amplified using ReactionReady PCR Mastermix (P1000A, SuperArray) from the pRS313 target plasmid (1) using the following primers.

URAtoHIS-1: 5’-CCT GCA GGA AAC GAA GAT AAA TCA TCA GAG CAG ATT GTA CTG AGA GTG CAC C-3’

URAtoHIS-2: 5’- GCT CTA ATT TGT GAG TTT AGT ATA CAT GCC GCA TCT GTG CGG TAT TTC ACA CCG C-3’

These primers contain flanking sequence homology to completely delete the *URA3* insert within the *GAL::*BRCA1 YEp24 plasmid. The resulting PCR product was introduced into the WT BY4741 strain containing the *GAL::*BRCA1-*URA3* plasmid by transformation. Following plating and selection on GLU-HIS 5FOA plates, the resulting His+ Ura- transformants were confirmed by PCR analysis to have the correct replacement of *URA3* with the *HIS3* marker leaving the BRCA1 gene unaffected. GAL-induced expression of BRCA1 (with selection for the *HIS3* marker) from this plasmid in the WT BY4743 strain resulted in lethality (see Fig. 1F in manuscript) similar to that seen with the parental *GAL::*BRCA1-*URA3* in the same strain.

Construction of the *GAL::*BRCA1_C-10_-*HIS3* expression plasmid. Using the approach described above, the *GAL::*BRCA1 plasmid *URA3* marker was replaced with *HIS3* amplified from pRS313. The primers used to amplify *HIS3* were URAtoHIS-1 described above and the following primer (C-10) that contains flanking homology to the C-terminus of BRCA1. Since the C-terminal end of BRCA1 is directly adjacent to the *URA3* marker, the C-10 primer inserts a stop codon to prematurely terminate and physically delete the final 33 base pairs of BRCA1 resulting in a BRCA1 protein that contains a C-terminal truncation of ten amino acids thereby eliminating BRCT function. BRCA1 C-terminal truncations longer than 8 amino acids interfere with BRCT folding and thus disrupt BRCA1 protein-protein interactions mediated through the BRCT domain. These mutations have been associated with early onset breast cancer (2).

C-10: 5’CTC TAC CAG TGC CAG GAG CTG GAC ACC TAC TGA CGC ATC TGT GCG GTA TTT CAC ACC GC-3’

Construction of the *GAL::*BRCT pDEST52 high copy expression plasmid. The sequence encoding the C-terminal 333 amino acids of BRCA1 was PCR amplified from the *GAL::*BRCA1 Yep24 expression plasmid using the following Gateway compatible primers and cloned into pDONR201 using BP clonase as per manufacturers instructions.

BRCT-L: 5’-GGG GAC AAG TTT GTA CAA AAA AGC AGG CTT CAC CCC TTA CCT GGA ATC TG-3’

BRCA1-R: 5’-GGG GAC CAC TTT GTA CAA GAA AGC TGG GTC CTA GTA GTG GCT GTG GGG GA-3’

Once established in pDONR201, the BRCT sequence was subcloned into pDEST52 and pDEST32 using LR clonase as per manufacturers instructions (Invitrogen). Expression of the BRCT sequence fused to the GAL4 DNA binding domain (in pDEST32) resulted in auto-transactivation of *HIS3* expression when established by transformation (along with the “empty” pEXP-AD502 GAL4 activation domain control plasmid) into the two-hybrid host strain MAV203 obtained in the Gateway compatible ProQuest two-hybrid interaction kit and assayed as per manufacturers instructions (Invitrogen, data not shown).

Construction of the *GAL::*BRCA1_M_ (I1766S)-*URA3* expression plasmid: A site specific mutation was placed within the BRCT domain of the *GAL::*BRCA1-*URA3* expression plasmid 5416 T to G (cDNA) I 1766 S using the Stratagene Qwikchange site-directed mutagenesis kit and the following forward and reverse primers as per manufacturers instructions.

Fwd: 5'- CAG GGG GCT AGA AAG CTG TTG CTA TGG GCC -3'

Rev: 5'- GGC CCA TAG CAA CAG CTT TCT AGC CCC CTG -3'

**Construction of the cyto-polII-CTD and nuc-polII-CTD high copy expression plasmids.** Both constructs encode L3-β-galactosidase-CTD fusion proteins on selectable (*URA3*) high copy YEp24 expression plasmids derived from the pLGSD5 parental vector (from H. Fried). The two constructs are similar with the exception that one contains the nuclear localization signal (NLS) from the *TCM1* gene that encodes the yeast ribosomal protein L3 fused in frame to the β-galactosidase gene and promotes the nuclear localization of the fusion protein product. The parental vector without the NLS (pTCM-RR) has been previously described (3). The parental vector with the NLS (pTCM-RA) is similar to pTCM-HA except that the *Rsa* I (-194) to Alu I (+91) fragment from *TCM1* was fused in frame to the β-galactosidase gene and contains the NLS (3). The complete yeast carboxy terminal domain from RNA polymerase II was subsequently cloned into each vector by inserting the 1.98 kb *Sst*I-*Eco*RI fragment from pURY-1 into pTCM-RR and pTCM-RA to produce pRRY1 (cyto-polII-CTD) and pRAY1 (nuc-polII-CTD) respectively. Each plasmid construct was sequenced to confirm integrity of the CTD fusion.

Construction of the *CTK1*-*HIS3* plasmid. The *CTK1-HIS3* plasmid (pJYC-1511) was created by cloning a 3.2 kb *Bgl*II-*Sal*I fragment carrying *CTK1* (from phage ZJ15 (4)) into the *Bgl*II/*Sal*I sites of pRS313.

**Construction of yeast strains expressing altered RNAPII Carboxy terminal domains.**   Rpb1p alleles with a C-terminal 10-histidine tags (His_10A_ ) were introduced by standard techniques onto the very C-terminus of Rpb1, as previously described (5). The 12AR Rpb1p mutant allele was a gift from J. Stiller. It was constructed as previously described (6) such that the wild type RNAPII CTD has been substituted with sequences that code for the repeated CTD sequence (YSPTSPSYSPTAAA)_6_ giving this truncated CTD allele a total of 12 heptapeptide repeats which is less than half the size of the normal wild type RNAPII CTD. All mutant Rpb1p alleles were contained within the Z26 yeast strain (7).

***DEF1-URA3* expression plasmid.** The *DEF1* expression plasmid was obtained as a yeast ORF clone within the selectable (*URA3*) expression plasmid BG1805 from Open Biosystems ([www.openbiosystems.com/GeneExpression/Yeast/ORF/](http://www.openbiosystems.com/GeneExpression/Yeast/ORF/)). The construction of these vectors has been previously described (8). The *DEF1* expression plasmid was established along with the *GAL::*BRCA1-HIS3 expression plasmid in the *def1Δ* diploid deletion strain by transformation. Co-expression of Def1p and BRCA1 in this strain re-established BRCA1-induced lethality (Fig. 1F).

**References**

1. Sikorski RS, Hieter P (1989) A system of shuttle vectors and yeast host strains designed for efficient manipulation of DNA in Saccharomyces cerevisiae. Genetics 122: 19-27.

2. Williams RS, Chasman DI, Hau DD, Hui B, Lau AY, et al. (2003) Detection of protein folding defects caused by BRCA1-BRCT truncation and missense mutations. J Biol Chem 278: 53007-53016.

3. Moreland RB, Nam HG, Hereford LM, Fried HM (1985) Identification of a nuclear localization signal of a yeast ribosomal protein. Proc Natl Acad Sci U S A 82: 6561-6565.

4. Lee JM, Greenleaf AL (1991) CTD kinase large subunit is encoded by CTK1, a gene required for normal growth of Saccharomyces cerevisiae. Gene Expr 1: 149-167.

5. Morris DP, Lee JM, Sterner DE, Brickey WJ, Greenleaf AL (1997) Assaying CTD kinases In vitro and phosphorylation-modulated properties of RNA polymerase II In vivo. METHODS, A companion to Methods in Enzymology 12: 264-275.

6. Stiller JW, Cook MS (2004) Functional unit of the RNA polymerase II C-terminal domain lies within heptapeptide pairs. Eukaryot Cell 3: 735-740.

7. West ML, Corden JL (1995) Construction and analysis of yeast RNA polymerase II CTD deletion and substitution mutations. Genetics 140: 1223-1233.

8. Gelperin DM, White MA, Wilkinson ML, Kon Y, Kung LA, et al. (2005) Biochemical and genetic analysis of the yeast proteome with a movable ORF collection. Genes Dev 19: 2816-2826.
